# Alveolar epithelial glycocalyx degradation mediates surfactant dysfunction and contributes to acute respiratory distress syndrome

**DOI:** 10.1172/jci.insight.154573

**Published:** 2022-01-25

**Authors:** Alicia N. Rizzo, Sarah M. Haeger, Kaori Oshima, Yimu Yang, Alison M. Wallbank, Ying Jin, Marie Lettau, Lynda A. McCaig, Nancy E. Wickersham, J. Brennan McNeil, Igor Zakharevich, Sarah A. McMurtry, Christophe J. Langouët-Astrié, Katrina W. Kopf, Dennis R. Voelker, Kirk C. Hansen, Ciara M. Shaver, V. Eric Kerchberger, Ryan A. Peterson, Wolfgang M. Kuebler, Matthias Ochs, Ruud A.W. Veldhuizen, Bradford J. Smith, Lorraine B. Ware, Julie A. Bastarache, Eric P. Schmidt

**Affiliations:** 1 Division of Pulmonary Sciences and Critical Care Medicine, Department of Medicine,; 2 Department of Bioengineering, and; 3 Department of Biostatistics and Informatics, School of Public Health, University of Colorado, Aurora, Colorado, USA.; 4 Institute of Functional Anatomy, Charité-Universitätsmedizin, Berlin, Germany.; 5 Department of Physiology and Pharmacology, Western University, London, Ontario, Canada.; 6 Department of Medicine and Department of Pathology, Microbiology and Immunology, Vanderbilt University, Nashville, Tennessee, USA.; 7 Department of Biochemistry and Molecular Genetics, University of Colorado, Aurora, Colorado, USA.; 8 Department of Medicine, National Jewish Health, Denver, Colorado, USA.; 9 Institute of Physiology, Charité-Universitätsmedizin, Berlin, Germany.; 10 Division of Pulmonary and Sleep Medicine, Department of Pediatrics, University of Colorado, Aurora, Colorado, USA.; 11 Department of Medicine, Denver Health Medical Center, Denver, Colorado, USA.

**Keywords:** Pulmonology, Glycobiology, Proteoglycans, Pulmonary surfactants

## Abstract

Acute respiratory distress syndrome (ARDS) is a common cause of respiratory failure yet has few pharmacologic therapies, reflecting the mechanistic heterogeneity of lung injury. We hypothesized that damage to the alveolar epithelial glycocalyx, a layer of glycosaminoglycans interposed between the epithelium and surfactant, contributes to lung injury in patients with ARDS. Using mass spectrometry of airspace fluid noninvasively collected from mechanically ventilated patients, we found that airspace glycosaminoglycan shedding (an index of glycocalyx degradation) occurred predominantly in patients with direct lung injury and was associated with duration of mechanical ventilation. Male patients had increased shedding, which correlated with airspace concentrations of matrix metalloproteinases. Selective epithelial glycocalyx degradation in mice was sufficient to induce surfactant dysfunction, a key characteristic of ARDS, leading to microatelectasis and decreased lung compliance. Rapid colorimetric quantification of airspace glycosaminoglycans was feasible and could provide point-of-care prognostic information to clinicians and/or be used for predictive enrichment in clinical trials.

## Introduction

Acute respiratory distress syndrome (ARDS) is a common cause of acute respiratory failure with substantial morbidity and mortality (1). Despite promising preclinical data, decades of clinical trials have been largely unsuccessful in identifying effective pharmacologic strategies (2). This disconnect has been increasingly attributed to mechanistic heterogeneity underlying this complex syndrome, inspiring efforts to identify ARDS subphenotypes that may impart differential responses to pharmacologic agents (3, 4). One commonly recognized determinant of ARDS heterogeneity is the route of the ARDS-triggering injury, differentiated between inhaled insults (“direct lung injury,” such as pneumonia or aspiration) and bloodstream-initiated pulmonary insults (“indirect lung injury,” such as sepsis or pancreatitis) (5). Despite the intuitive differences between these forms of ARDS, their mechanistic underpinnings are not well understood, and these crude classifications likely oversimplify complex underlying pathophysiology. A thorough understanding of the patient-level factors and mechanisms driving ARDS heterogeneity is critical for the development and implementation of precision strategies for ARDS therapy.

Direct pulmonary insults are posited to cause ARDS by damaging the alveolar epithelium and key supporting structures, including pulmonary surfactant, a complex mixture of phospholipids and proteins that lines the alveolar airspace and serves to prevent alveolar collapse during tidal breathing (6). Surfactant is separated from the alveolar epithelium by a glycosaminoglycan-enriched epithelial glycocalyx that, despite having been recognized for over 50 years, is understudied (7, 8). The uncertainty regarding the homeostatic function of the alveolar epithelial glycocalyx contrasts with a robust literature investigating the pulmonary endothelial glycocalyx during health and disease (9–12). Our laboratory has recently described that the alveolar epithelial glycocalyx is damaged in murine models of direct lung injury, resulting in increased alveolocapillary permeability and shedding of glycocalyx fragments into the airspace (8, 13). However, the role of the alveolar epithelial glycocalyx in human ARDS has not been investigated.

We sought to determine the contribution of alveolar epithelial glycocalyx degradation to ARDS heterogeneity and pathogenesis. We hypothesized that airspace fluid glycosaminoglycan (GAG) concentrations would correlate with lung injury severity and disease course. Additionally, given the intimate anatomic and proposed functional relationship between the epithelial glycocalyx and pulmonary surfactant, we also hypothesized that glycocalyx degradation would mediate lung injury by disrupting surfactant function independently of damage to alveolar epithelial cells. Finally, we sought to determine the feasibility of point-of-care quantification of glycocalyx degradation in mechanically ventilated patients, enabling airspace GAG shedding to be used to inform prognosis at the bedside and/or for predictive enrichment in clinical trials of ARDS therapeutics.

## Results

### Noninvasive detection of alveolar epithelial glycocalyx degradation in respiratory failure.

**Figure 1:**
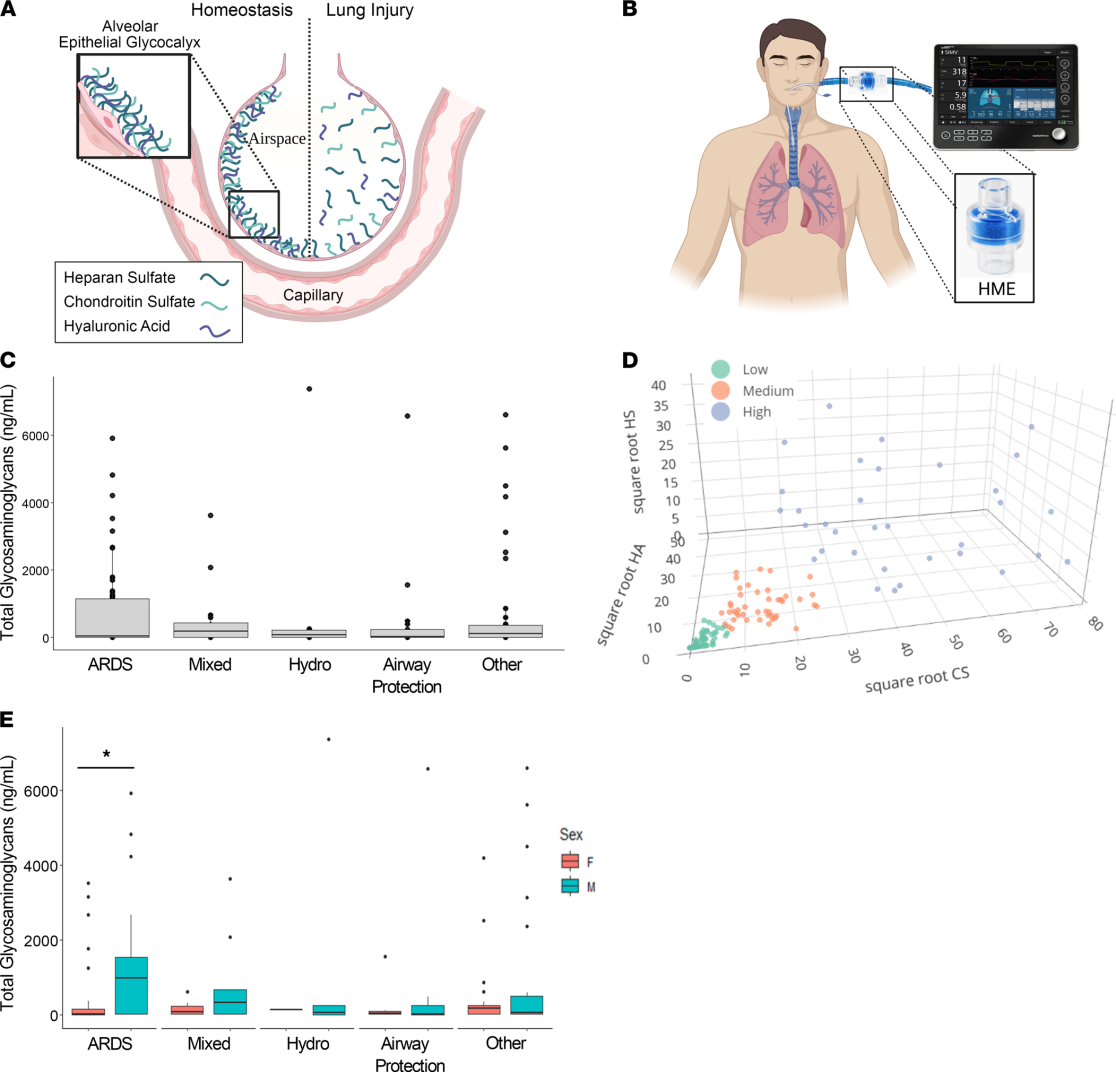
GAG shedding is heterogeneous in ARDS. (**A**) Graphical representation (not to scale) of the alveolar epithelial glycocalyx, a layer of GAGs — heparan sulfate (HS), chondroitin sulfate (CS), and hyaluronic acid (HA) — that lines the apical alveolar epithelial surface. In murine models of acute lung injury, the glycocalyx is damaged, leading to shedding of GAGs into the airspace fluid. (**B**) Graphical representation of a patient on a ventilator circuit containing a heat and moisture exchanger (HME) filter, from which airspace fluid can be noninvasively sampled in mechanically ventilated patients. (**C**) Assessment of airspace fluid GAG shedding in mechanically ventilated patients was performed by mass spectrometry analysis of HMEF. (**D**) Partitioning around k-medoids clustering using the variables HS, CS, and HA was performed to group patients into high, medium, and low shedding clusters based on the degree of GAG shedding. (**E**) Assessment of the effect of patients’ sex on airspace GAG levels in patients with respiratory failure. *n* = 153 participants with respiratory failure. *P < 0.05 by Wilcoxon’s rank sum test. Data are represented as median and IQR with individual data points for those outside of 1.5 × IQR. **A** and **B** were created with BioRender (biorender.com).

The alveolar epithelial glycocalyx, a complex layer of GAGs that lines the apical epithelial surface, is damaged in murine models of lung injury, leading to shedding of GAG fragments into the bronchoalveolar lavage (BAL) (Figure 1A) (8, 13). Given the tenuous clinical status of patients with ARDS, bronchoscopy to obtain BAL is infrequently performed, resulting in a scarcity of airspace samples for translational ARDS research (4). Furthermore, clinically obtained bronchoscopic samples may be confounded by variability in airspace fluid dilution during BAL. In contrast to BAL, airspace fluid can be collected noninvasively from mechanically ventilated patients with ARDS using heat and moisture exchanger (HME) filters, bacteriostatic sponges that are routinely used in the care of mechanically ventilated patients (Figure 1B). The contents of HME fluid (HMEF) closely approximate the contents of directly aspirated (and thus undiluted) pulmonary edema fluid, thus providing a novel and noninvasive method of sampling the distal airspaces in mechanically ventilated patients (14, 15).

We conducted a prospective observational cohort study of 153 mechanically ventilated intensive care unit (ICU) patients at Vanderbilt University Medical Center to identify clinical factors associated with GAG shedding into HMEF. The study population included 66 participants with ARDS (per the 2012 Berlin Definition; ref. 16), 6 participants with cardiogenic pulmonary edema (“Hydro” in the figures), 15 participants who were classified as having “mixed” cardiogenic and noncardiogenic pulmonary edema, 21 participants who were intubated for airway protection in the absence of lung disease, and 40 participants with respiratory failure due to other causes. HMEF was collected within 24 hours of initiation of mechanical ventilation. Descriptive statistics of the patients are presented in Table 1. State-of-the-art liquid chromatography–tandem mass spectrometry multiple reaction monitoring, optimized for the detection of GAGs in biologic specimens (17), revealed a wide range of GAG concentrations in HMEF (0–7395 ng/mL). Within each etiology of respiratory failure there was a wide range in the degree of GAG shedding, particularly in patients with ARDS (median 34.37 ng/mL, IQR 1148 ng/mL) (Figure 1C). There was no association between GAG shedding and chronic medical comorbidities, suggestive that epithelial glycocalyx degradation occurs as an acute process in the setting of respiratory failure, rather than constitutively due to chronic illness (Supplemental Figure 1; supplemental material available online with this article; https://doi.org/10.1172/jci.insight.154573DS1). Additionally, HMEF GAG concentrations were independent of minute ventilation, respiratory rate, tidal volume, tidal volume adjusted for ideal body weight, and positive end expiratory pressure (Supplemental Figure 2), demonstrating that variability in GAG shedding was not an artifact of differences in airflow across the HME filter.

In order to determine the patient-level factors driving the observed heterogeneity in GAG shedding, we performed partitioning around medoids (PAM; or k-medoid) clustering analysis to identify patients with low, medium, and high GAG shedding using each of the individual GAG species — chondroitin sulfate (CS), heparan sulfate (HS), and hyaluronic acid (HA) — as the input variables (Figure 1D). We found that male patients were more likely to be in the high GAG shedding group compared with females (median total GAGs of 158.40 ng/mL for males vs. 31.83 ng/mL for females, *P* = 0.018); however, there were no significant associations between GAG shedding cluster and the other demographics, including age, race, ethnicity, BMI, and smoking status (Table 2). Interestingly, sex differences in GAG shedding were driven by the ARDS group (median total GAGs of 974.48 ng/mL for males vs. 11.64 ng/mL for females, *P* = 0.0031), and there were no statistically significant differences in GAG shedding based on sex in the other types of respiratory failure (Figure 1E). Statistical models of HMEF GAG shedding including an interaction between sex and age did not identify a sex-specific age effect, and the sex difference remained significant after adjusting for the effect of age, suggesting that it is not mediated (nor modified) by a protective effect of estrogen signaling in younger women (Supplemental Figure 3).

### Alveolar epithelial glycocalyx degradation occurs in patients with direct lung injury and is associated with upregulation of matrix metalloproteinases.

**Figure 2:**
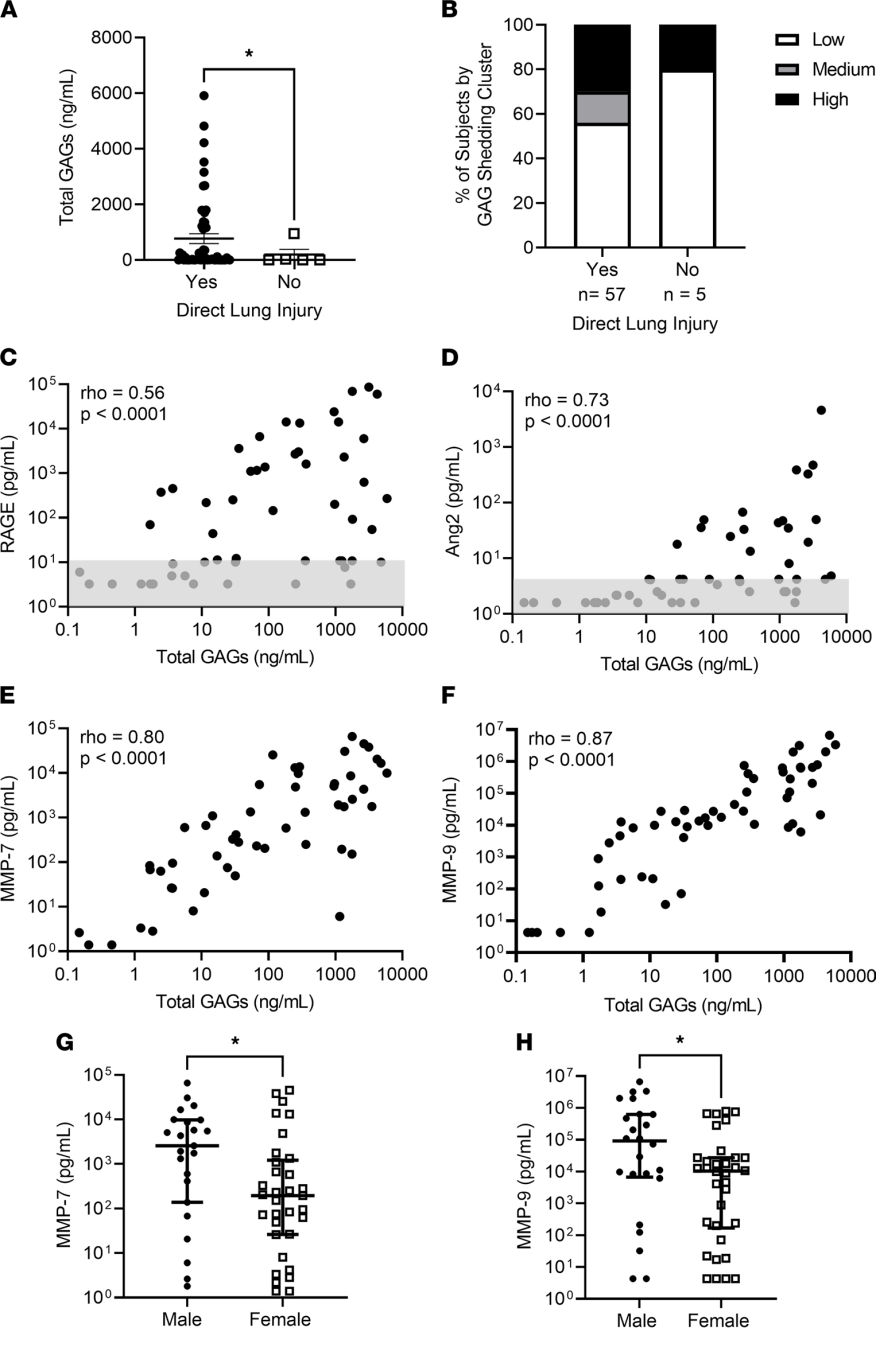
Alveolar epithelial glycocalyx degradation occurs in patients with direct lung injury and is associated with upregulation of matrix metalloproteinases. (**A**) Assessment of total GAG shedding by clinically determined risk factor for lung injury. Direct lung injury indicates patients who were diagnosed with either pneumonia or aspiration at the time of intubation. *n* = 62 patients with ARDS. *P* = 0.045 by Kruskal-Wallis test. Data are represented as mean ± SEM. (**B**) Assessment of the percentage of patients in each GAG shedding cluster for patients with and without direct lung injury. (**C**–**F**) Assessment of the relationship between GAG shedding and the epithelial cellular injury marker receptor for advanced glycation end products (RAGE) (*n* = 56) (**C**), the endothelial cellular injury marker angiopoietin-2 (Ang2) (*n* = 56) (**D**), and the matrix metalloproteinases MMP-7 (*n* = 56) (**E**) and MMP-9 (*n* = 58) (**F**). Gray boxes (**C** and **D**) represent values at or below the lower limit of detection (variability of this limit reflects different sample dilutions). Spearman’s ρ and *P* values are as indicated on each graph. (**G** and **H**) Assessment of the airspace MMP-7 (**G**) and MMP-9 (**H**) levels based on sex. *P* values were computed by Wilcoxon’s rank sum test. *P < 0.05. Data are represented as mean ± SEM.

Based on the location of the epithelial glycocalyx at the inner lining of the alveoli, we hypothesized that its degradation would occur predominantly in patients with direct causes of lung injury and that the type of lung injury may contribute to the observed heterogeneity in airspace GAG shedding (5, 18). Participants with ARDS who had risk factors for direct lung injury (pneumonia or aspiration) had increased GAG shedding compared with those who only had risk factors for indirect lung injury (sepsis, pancreatitis, transfusion, or trauma) (*P* = 0.046) (Figure 2A). Interestingly, although 96% of the patients with ARDS who were in the medium and high shedding clusters had risk factors for direct lung injury, 56% of the patients with ARDS with direct lung injury were in the low shedding cluster, suggesting that glycocalyx degradation only occurs in a subset of patients with direct lung injury and may be a source of ARDS heterogeneity (Figure 2B). Levels of the lung epithelial injury marker receptor for advanced glycation end products (RAGE), which is frequently used as a measure of direct lung injury in ARDS, were associated with airspace GAG levels (Spearman’s ρ = 0.56, *P* < 0.0001) (Figure 2C) (19). Airspace levels of the endothelial cellular injury marker angiopoietin-2 (Ang2) were very low until high levels of GAG shedding, which is likely attributable to increased vascular permeability with high levels of epithelial injury (Figure 2D) (20). Airspace GAG concentrations did correlate with IgM, a serum protein with little penetration into the alveolar airspace during health; however, there was no correlation between airspace GAG levels and the radiographic assessment of lung edema (RALE) score, a method that uses the chest radiograph to quantify pulmonary edema. As the RALE score highly correlates with lung weight, airspace GAG shedding is likely not solely a consequence of pulmonary edema (Supplemental Figure 4).

Previous studies using murine lung injury models have demonstrated that epithelial glycocalyx degradation occurred as a result of upregulation of matrix metalloproteinases (MMPs), proteases that cleave proteoglycans anchoring GAGs to the epithelial surface (8, 13). MMP-7 and MMP-9 are known to cleave HS proteoglycans (including syndecan-1) and have been implicated in epithelial glycocalyx degradation in LPS- and bleomycin-induced lung injury (8, 13, 21, 22). We identified a strong correlation between airspace GAGs and MMP-7 (ρ = 0.80, *P* < 0.0001) and MMP-9 (ρ = 0.87, *P* < 0.0001) (Figure 2, E and F). Additionally, among patients with ARDS, both MMP-7 and MMP-9 levels were higher in males than females, potentially explaining the aforementioned sex differences in HMEF GAG shedding (Figure 2, G and H). While our prior animal work suggests that MMPs are causative in epithelial glycocalyx degradation, we have not investigated MMP activation in HMEF given that it is unclear whether the filter itself may induce activation of these proteins (8, 13).

### Alveolar epithelial glycocalyx degradation correlates with ARDS severity and duration.

**Figure 3:**
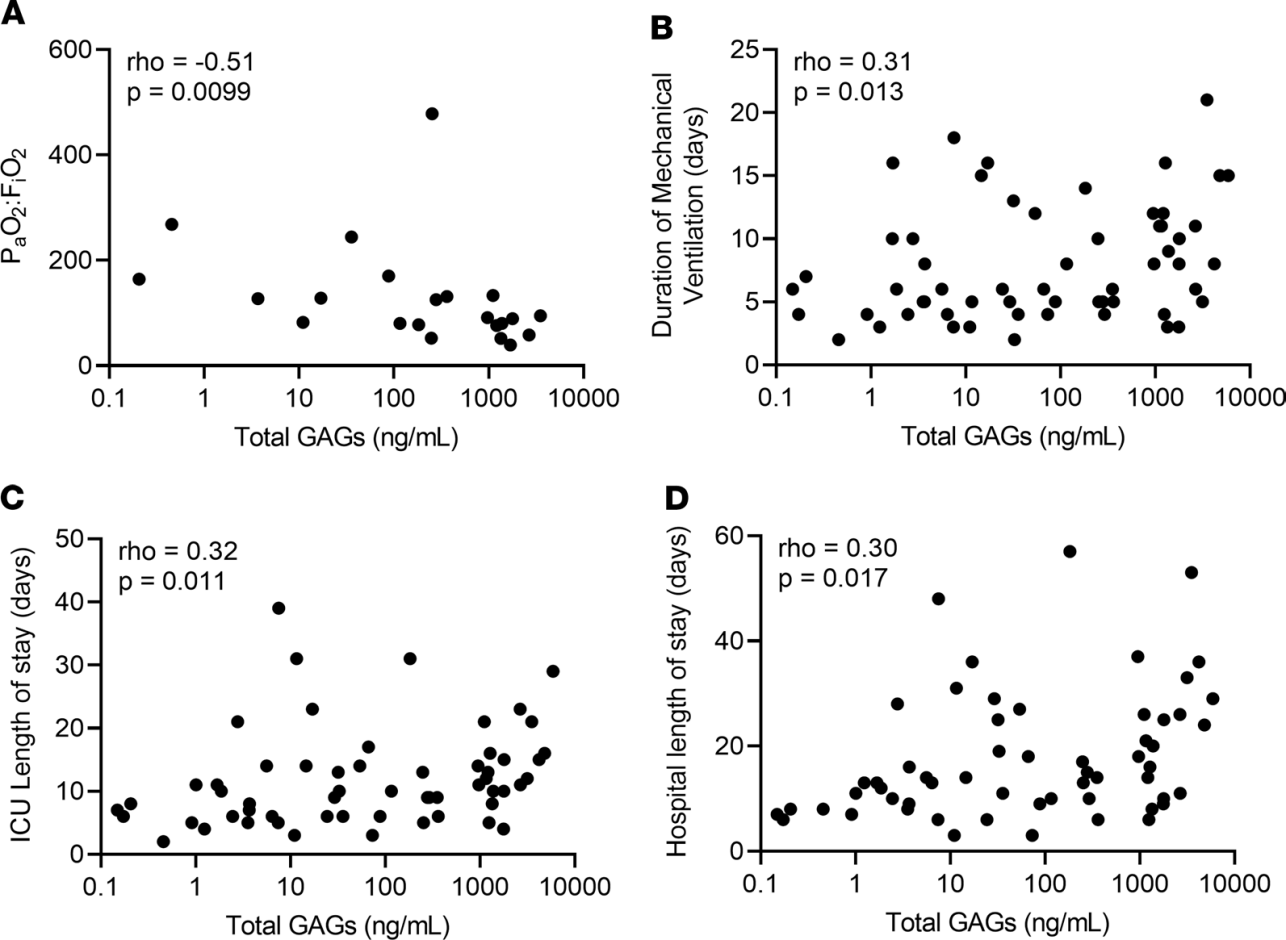
GAG shedding predicts ARDS severity and duration. (**A**) Assessment of the relationship between GAG shedding and P_a_O_2_:F_i_O_2_. *n* = 25 patients with ARDS in whom arterial blood gas data were available. (**B**–**D**) Assessment of the relationship between GAG shedding and duration of mechanical ventilation (**B**), ICU length of stay (**C**), and hospital length of stay (**D**) in patients with ARDS. *n* = 64 patients with ARDS in whom full data regarding duration of critical illness were available. Spearman ρ and *P* values were computed as indicated on each graph.

We next sought to evaluate the prognostic relevance of glycocalyx degradation in ARDS by determining the relationship between GAG shedding and clinical outcomes in the subgroup with ARDS (*n* = 66). Bivariate analysis demonstrated that the quantity of GAG shedding was closely associated with degree of hypoxemia (P_a_O_2_:F_i_O_2_) where P_a_O_2_ is defined as the partial pressure of arterial oxygen and F_i_O_2_ is the fraction of inspired oxygen (ρ = –0.51, *P* = 0.0099) in patients with ARDS at the time of HME collection (Figure 3A). Additionally, GAG shedding (at day 1 of mechanical ventilation) was associated with the subsequent duration of critical illness, as quantified by total days of mechanical ventilation (ρ = 0.31, *P* = 0.013), ICU length of stay (ρ = 0.32, *P* = 0.011), and hospital length of stay (ρ = 0.30, *P* = 0.017) in patients with ARDS (Figure 3, B–D).

Furthermore, in analyses that included the entire cohort of patients with respiratory failure, the associations between degree of GAG shedding and clinical outcomes were robust to adjustment for age, sex, race, and BMI using multivariable proportional odds ordinal regression models. Compared with participants in the low shedding group, participants in the high shedding group had 4.14 times greater odds of spending more days on mechanical ventilation (95% CI = 1.08–15.91, *P* = 0.04). Similarly, the subset of 25 patients with ARDS who had an arterial blood gas drawn as part of usual clinical care at the time of filter collection revealed that patients in the high shedding group had worsened hypoxemia (decreased P_a_O_2_:F_i_O_2_) compared with patients in the low shedding group: participants in the high shedding group had 9.34 greater odds of having a lower P_a_O_2_:F_i_O_2_ compared with those in the low shedding group (95% CI = 1.19–71.4, *P* = 0.03) (Table 3). These multivariable proportional odds ordinal regression models also demonstrated a trend toward an association between high GAG shedding and increased ICU days (OR = 2.46, 95% CI = 0.68–8.97, *P* = 0.17) and hospital days (OR = 3.72, 95% CI = 0.97–14.25, *P* = 0.06) compared with the low shedding group.

In a multivariable logistic regression model, there was no association between GAG shedding cluster and mortality in ARDS or the entire cohort of mechanically ventilated patients. The adjusted OR of death in the high shedding group versus the low shedding reference group was 0.43 (95% CI = 0.01–1.67, *P* = 0.23) for the subgroup with ARDS and 0.53 (95% CI = 0.20–1.30, *P* = 0.17) for the entire cohort, suggestive that the relationship between GAG shedding and days on mechanical ventilation was not confounded by mortality. Given that airspace GAG concentrations were determined using HMEF collected within 24 hours of intubation, these findings may be useful for the care of ICU patients, as there are few clinically available tools to predict the duration of respiratory failure at the bedside (23).

### Alveolar epithelial glycocalyx degradation induces microatelectasis by impairing surfactant function.

**Figure 4:**
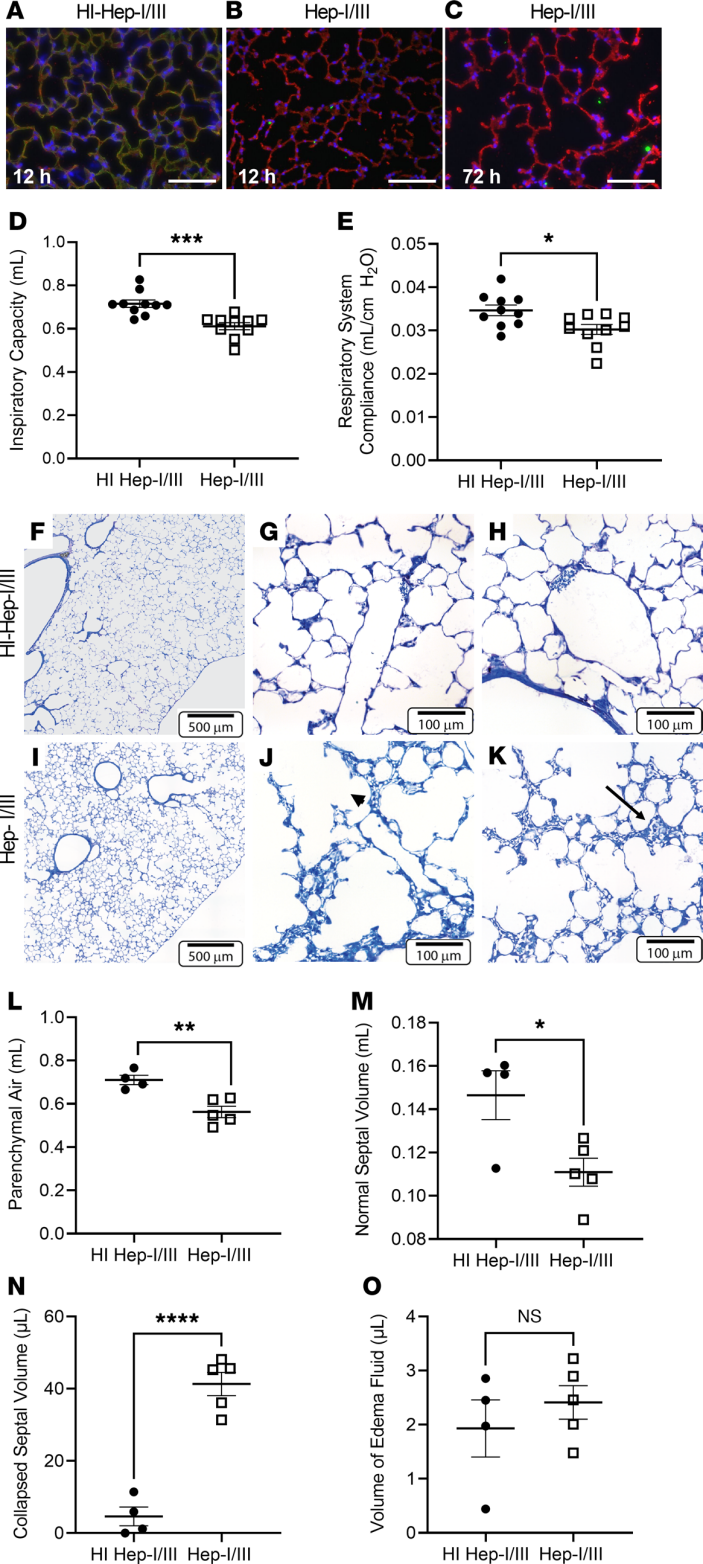
Alveolar epithelial glycocalyx degradation impairs lung compliance by causing microatelectasis. (**A**–**C**) After treatment with heat-inactivated heparinases I/III (HI Hep-I/III; 15 U, intratracheal), mouse lungs demonstrated colocalization of HS (HS 10E4, green) and the alveolar epithelium (*Lycopersicon esculentum* agglutinin lectin, red). Mice treated with active heparinases I/III (Hep-I/III, 15 U, intratracheal) demonstrated a denuded epithelial layer without HS staining at 12 hours, which persisted at 72 hours. As lungs were not perfusion-fixed, the endothelial glycocalyx is not visible. Scale bars: 100 μm. (**D** and **E**) Assessment of the lung mechanics of mice treated with Hep-I/III and HI Hep-I/III at *t* = 24 hours. *n* = 10 mice per group. Unpaired Student’s *t* test was used for comparisons between 2 groups. *P < 0.05, ***P < 0.0005. (**F**–**K**) Lung histology of Hep-I/III– and HI Hep-I/III–treated mice (24 hours). Dilated alveolar ducts (arrowhead) and microatelectasis (arrow) were noted at increased frequency in Hep-I/III–treated mice. (**L**–**O**) Quantification of the histologic changes present in Hep-I/III–treated mice as assessed by unbiased stereologic assessment of the lung architecture. There was no evidence of fibrosis in either group. *n* = 4–5 mice per group. Unpaired *t* test was used for comparisons between 2 groups. *P < 0.05, **P < 0.005, ****P < 0.0001. Data are represented as mean ± SEM.

Given the relationship between GAG shedding and clinical outcomes in patients with ARDS, we next sought to determine the attributable effect of alveolar epithelial glycocalyx degradation on lung injury pathogenesis using animal models. We performed intratracheal injections of heparinase I/III (Hep-I/III, 15 U), an HS-specific bacterial glucuronidase (24), or heat-inactivated (HI) Hep-I/III (negative control) in male C57BL/6 mice (Figure 4, A–C) (8). We chose male mice to mimic the male predominance of GAG shedding observed in HME filters from humans with ARDS (Figure 1E). Hep-I/III specifically targeted airspace HS but also induced secondary release of other GAGs (such as CS), thereby reproducing our HME findings (Supplemental Figure 5, A–D). We previously demonstrated that intratracheal heparinases cause epithelial glycocalyx degradation without inducing lung inflammation or edema (8), suggesting that these enzymes do not directly injure epithelial cells. Indeed, intratracheal Hep-I/III did not increase BAL cell counts or lactate dehydrogenase, a marker of cell death (Supplemental Figure 5, E and F) (25). Furthermore, although Hep-I/III–treated mice had increased BAL protein, the epithelial permeability of intratracheally administered labeled albumin into the circulation was unchanged after the administration of Hep-I/III, suggesting that epithelial viability was unharmed by glycocalyx degradation (Supplemental Figure 5, G and H).

Interestingly, despite the absence of pulmonary edema (8), Hep-I/III–treated mice demonstrated decreased inspiratory capacity and impaired lung compliance 24 hours after injection (Figure 4, D and E). These changes were mediated specifically by HS, as mice that were treated with intratracheal chondroitinase ABC, a model that our laboratory has previously used to degrade epithelial surface CS (8), did not have impaired pulmonary mechanics compared with those treated with heat-inactivated chondroitinases (Supplemental Figure 6). Unbiased stereologic quantification of the lung architecture of Hep-I/III–treated mice revealed the induction of microatelectasis (defined by areas in which 2 or more layers of alveolar capillaries are directly opposed to each other, indicating alveolar collapse) (26) in Hep-I/III–treated mice (Figure 4, F–N). There was minimal alveolar edema in either group (Figure 4O). Structure-function analyses demonstrated a strong correlation between the degree of microatelectasis and the lung mechanics abnormalities, suggestive that microatelectasis mediates the observed loss of lung compliance (Supplemental Figure 7). Micro-CT scans of these animals confirmed lower lung volumes and a shift of the average Hounsfield units throughout the lung toward decreased aeration, suggestive of a subtle but widespread process, such as microatelectasis (Supplemental Figure 8).

**Figure 5:**
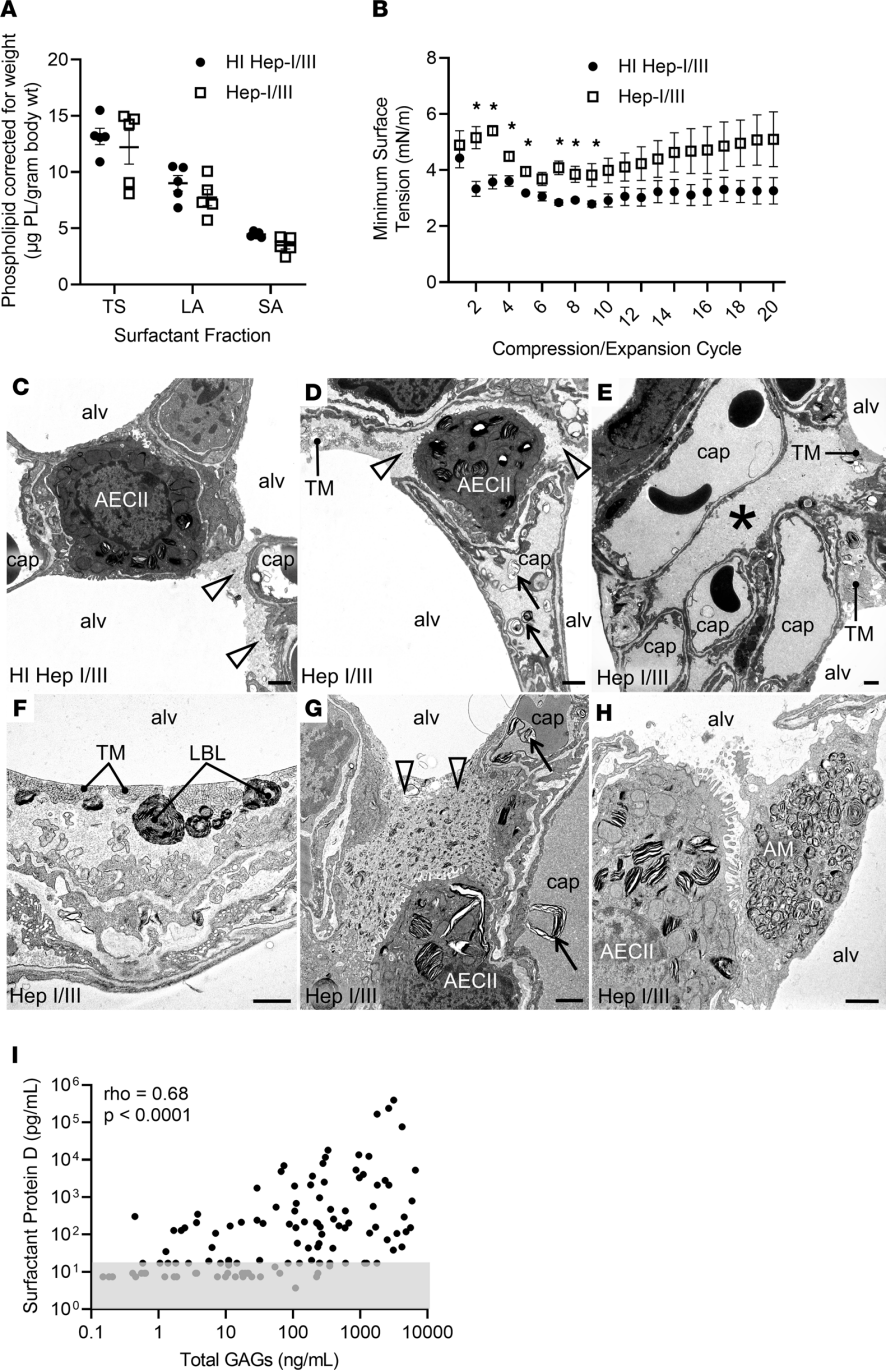
Alveolar epithelial glycocalyx degradation impairs surfactant function. (**A**) The effect of Hep-I/III treatment on the quantity of total surfactant (TS) and its subfractions, the surface-active large aggregates (LA) and the inactive small aggregates (SA). (**B**) The minimum surface tension of the LA surfactant subfraction from Hep-I/III–treated mice compared with HI Hep-I/III–treated controls. *n* = 5 mice per group. *P < 0.05 by 2-tailed unpaired *t* test. Data are represented as mean ± SEM. (**C**–**H**) In both HI Hep-I/III–treated (**C**) and Hep-I/III–treated (**D**–**H**) animals, transmission electron microscopy revealed a thin layer of electron-dense alveolar liquid film (open arrowheads) containing lipid fragments on top of the alveolar epithelium. This film had an increased thickness in Hep-I/III–treated mice (**D**). Higher amounts were particularly found in alveolar corners and interalveolar pores of Kohn or covering the secretory surface of type II alveolar epithelial cells (AECII; **C**, **D**, **G**, and **H**). Alveolar liquid–filled infoldings of the alveolar epithelium (asterisk) were present to a larger extent in the Hep-I/III–treated mice (**E**). In Hep-I/III–treated mice, intra-alveolar surfactant subtypes, in particular freshly secreted lamellar body–like forms (LBL) in close proximity to tubular myelin (TM) at the air-liquid interface, were more abundant (**F**). The electron density of the liquid film and the quantity of contained small lipid fragments were increased in the Hep-I/III group (**G**). Lamellar lipid accumulations were also found in some alveolar macrophages (AM; **H**) and capillaries (arrows; **D** and **G**). alv, alveolar lumen; cap, capillary lumen. Scale bars: 1 μm. (**I**) The relationship between GAG shedding and surfactant protein D in HMEF. Gray box represents values at or below the lower limit of detection (variability of this limit reflects different sample dilutions). *n* = 115 participants with respiratory failure. Spearman ρ and *P* values were calculated as indicated.

Pulmonary surfactant, a mixture of lipids and proteins that is secreted by the alveolar type II epithelial cells into the alveolar space, reduces surface tension at the air-liquid interface of the lung and thus prevents alveolar collapse and microatelectasis during exhalation (27). Although the epithelial glycocalyx is interposed between surfactant and the apical epithelial surface, the functional relationship between surfactant and the epithelial glycocalyx is unknown (7). We found that the BAL total surfactant quantity was similar in Hep-I/III– and HI Hep-I/III–treated mice, as were the relative amounts of active surfactant (large aggregates) and inactive surfactant (small aggregates) (Figure 5A). Additionally, thin-layer chromatography confirmed that BAL from Hep-I/III–treated mice did not have alterations in the relative contribution of individual surfactant phospholipid species (Supplemental Figure 9). However, constrained sessile drop surfactometry analysis of surfactant isolated from the BAL of Hep-I/III–treated mice demonstrated higher minimum surface tension in comparison with HI Hep-I/III–treated mice, indicative that glycocalyx degradation impairs surfactant function (Figure 5B). This increase in alveolar surface tension is modest in contrast to the substantial injury with massive degrees of injury; however, even small increases in surface tension are sufficient to contribute to lung injury and could make the lung more susceptible to secondary insult (28–30).

The observed impairment in surfactant function was mediated by the loss of epithelial surface GAGs, rather than the presence of shed GAGs within the airspace, as the addition of exogenous HS or CS (at concentrations similar to what is shed into the alveolar lining fluid after glycocalyx degradation) to the BAL of uninjured mice did not impair surfactant function (Supplemental Figure 10, A and B). Furthermore, Hep-I/III–treated mice continued to display impaired lung compliance at 72 hours, a time point at which GAG fragments were cleared from the airspace (Supplemental Figure 10, C and D) yet the loss of epithelial surface GAGs persisted (Figure 4C).

Surfactant production and turnover of dysfunctional surfactant are constitutive processes with dysfunctional surfactant being removed from the air-liquid interface via uptake into alveolar macrophages and epithelial cells (31). In accordance with our in vivo observation of glycocalyx degradation inducing surfactant dysfunction, ultrastructural analysis using transmission electron microscopy revealed that epithelial glycocalyx degradation alters surfactant integrity and turnover. Ultrastructural alterations in Hep-I/III–treated mice included discrete areas of high–electron density (likely high-protein) fluid within the alveolar corners and over interalveolar pores of Kohn, occasional infoldings of the alveolar epithelium filled with electron-dense liquid, and an accumulation of lamellar lipid material in capillaries and alveolar macrophages (Figure 5, C–H), suggesting increased removal of dysfunctional surfactant from the distal airspaces. RNA sequencing of the whole-lung homogenate of mice treated with Hep-I/III demonstrated a subtle increase in expression of surfactant protein D (SP-D), which may also reflect increased surfactant turnover (Supplemental Figure 11).

These alterations in surfactant function and fate suggest a critical structural interaction between alveolar epithelial glycocalyx GAGs (such as HS) and pulmonary surfactant at the epithelial surface. The length and disaccharide sulfation patterns of HS polysaccharides determine their ability to interact with proteins via electrostatic interaction, akin to heparin (a highly sulfated form of HS) binding to antithrombin III (12). We previously found that epithelial HS shed into the airspace after direct lung injury shares size and sulfation patterns similar to those of heparin (13). Thus, to screen which surfactant protein(s) are capable of binding shed HS fragments, we performed heparin affinity chromatography using the BAL of male and female mice collected 7 days after infection with PR8 influenza virus (Supplemental Figure 12). Unbiased proteomic mass spectrometry identified that the heparin binding fraction contained SP-A, -B, and -D. These in vivo findings linking GAG degradation with surfactant dysfunction are likely relevant to human disease, as HMEF GAGs correlated with SP-D levels (ρ = 0.68, *P* < 0.0001) (Figure 5I). Together these data suggest that the glycocalyx may play a role in the localization and stabilization of surfactant proteins (and thus surfactant) at the air-liquid interface.

### Feasibility of point-of-care quantification of airspace GAGs in the ICU.

**Figure 6:**
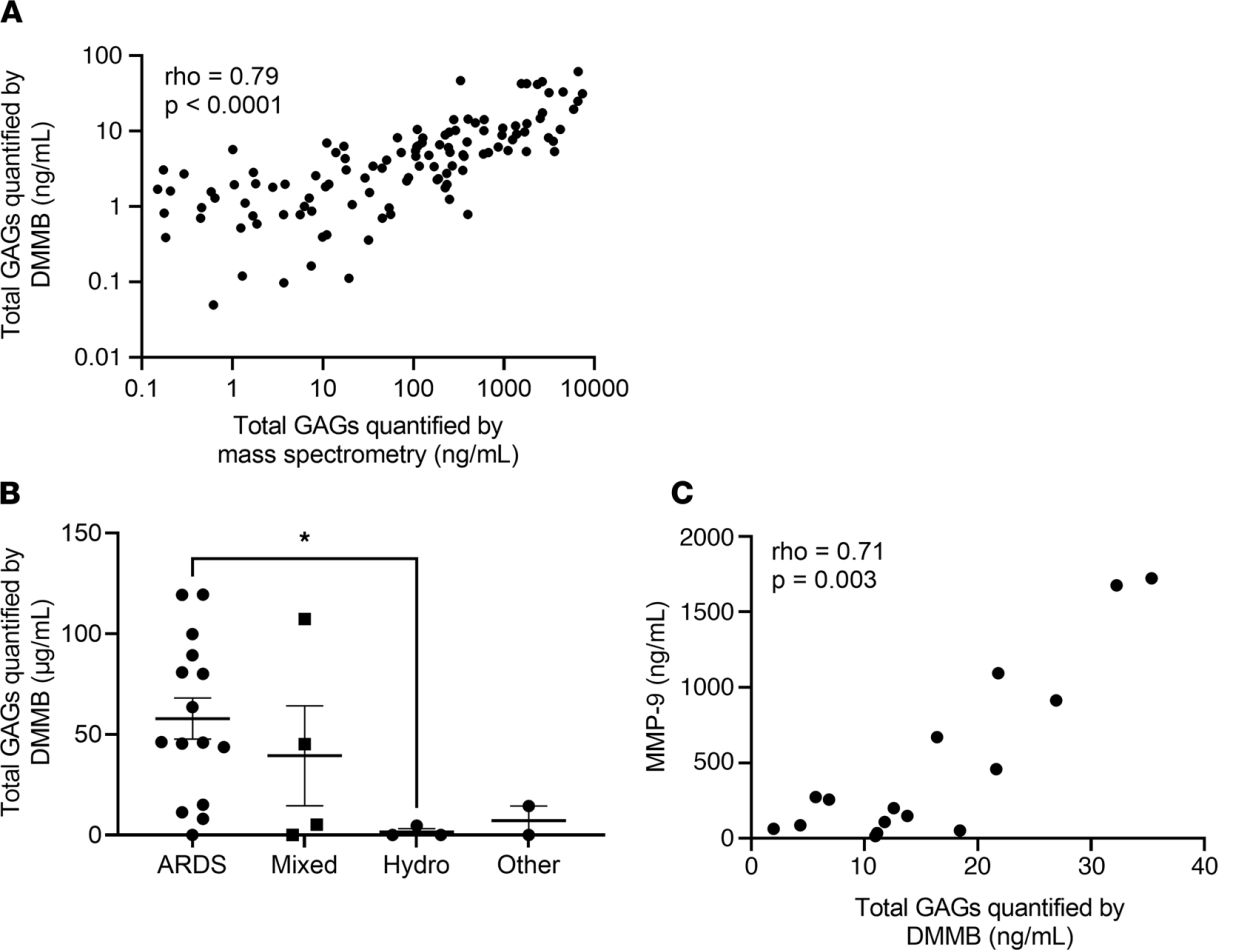
Point-of-care detection of alveolar epithelial glycocalyx degradation approximates mass spectrometry and is feasible in the ICU. (**A**) Assessment of the relationship between GAG shedding as quantified by state-of-the-art HPLC-MS and GAG shedding as quantified by colorimetric DMMB assay. *n* = 132 participants in whom sufficient fluid was available to run the DMMB assay. Spearman ρ and *P* values are as indicated on the graph. (**B**) Assessment of the relationship between GAG shedding by DMMB assay and cause of respiratory failure in a second cohort of patients in whom filters were collected only at routine filter changes, rather than through a standardized research study protocol. *n* = 24 participants with respiratory failure. *P < 0.05 by Wilcoxon’s rank sum test. Data are presented with mean ± SEM. (**C**) Assessment of the relationship between GAG shedding by DMMB assay and MMP-9 expression in the cohort of patients with filters collected only as part of routine care. *n* = 16 participants with respiratory failure. Spearman ρ and *P* values are as indicated on the graph.

Given that mass spectrometry is expensive and cannot be easily performed at the bedside, we sought to determine the predictive value of dimethylmethylene blue (DMMB), a rapid colorimetric assay of sulfated GAGs. While we have previously demonstrated that DMMB detects sulfated GAGs in urine and predicts acute kidney injury (32), the utility of DMMB measurements of airspace fluid is unexplored. We found that DMMB closely approximated mass spectrometry quantification of total GAG levels in HMEF (ρ = 0.79, *P* < 0.0001) (Figure 6A). To determine whether this approach is sufficiently robust to allow for practical clinical sampling techniques outside of the confines of a standardized research protocol, we collected airspace fluid from an additional cohort of 24 patients in whom HME filters were collected only as part of standard clinical filter exchanges (variable filter dwell times in the ventilator circuit). Similar to the initial cohort, in which HME filters were collected under a standardized collection protocol, HME filters collected after routine clinical use from patients with ARDS demonstrated elevated and heterogeneous GAG shedding compared with hydrostatic pulmonary edema controls (Figure 6B). Furthermore, GAG levels using simplified sample collection also tightly correlated with MMP-9 (ρ = 0.71, *P* = 0.003) (Figure 6C). Together, these data support the real-world utility and feasibility of quantification of airspace GAGs using HMEF.

## Discussion

Our bedside-to-bench study demonstrates that alveolar epithelial glycocalyx integrity is critical to surfactant function and that shedding of this layer is associated with the duration of respiratory failure in patients with ARDS and all-cause respiratory failure. Consistent with the known heterogeneity of ARDS, we observed substantial heterogeneity of airspace GAG shedding, with increased shedding associated with male sex and direct lung injury. To determine the specific impact of GAG shedding in the absence of epithelial cell death, we used a murine model of selective degradation of the alveolar epithelial glycocalyx. This model demonstrated that alveolar epithelial glycocalyx degradation impaired pulmonary mechanics in the absence of inflammation and pulmonary edema. This decreased lung function correlated with (and was likely mediated by) microatelectasis, which occurred in the setting of surfactant dysfunction. Based on these findings, we speculate that pharmacologic agents aimed at either blocking glycocalyx degradation or enhancing native mechanisms of regeneration could be used in patients with either risk factors for GAG shedding (male sex and pneumonia/aspiration) or elevated HMEF GAG levels, as assessed by DMMB.

Our data reveal a significant difference in glycocalyx degradation based on sex that was largely driven by the subset of patients with ARDS. These findings were not an artifact of sex-based differences in minute ventilation (and thus flow across the HME filter). Sex-based differences did not vary according to age, suggesting that these effects are not mediated by a protective effect of estrogen, which would be expected to diminish after menopause. Sex differences in airspace GAG levels correlated with sex-based differences in MMP expression, a phenomenon that has been reported in other disease states including cardiovascular disease (33, 34). Epidemiologic studies have reported mixed results as to whether sex is an independent risk factor for ARDS (35, 36); however, these studies often included a wide heterogeneity of ARDS risk factors, including patients with indirect lung injury in whom minimal epithelial glycocalyx degradation would be expected. Interestingly, sex differences in outcomes are well established in coronavirus disease 2019 (COVID-19), an ARDS population that is uniquely enriched in direct lung injury (37). Additional studies are necessary to determine whether severe acute respiratory syndrome coronavirus 2 (SARS-CoV-2) infection induces glycocalyx degradation, as COVID-19 patients were not included in the present study owing to biosafety concerns with HME filter handling.

Dysfunction of pulmonary surfactant has long been recognized as a key contributor to ARDS pathophysiology (6); however, the mechanisms responsible for this dysfunction remain under active investigation. Given their intimate anatomic relationship, we hypothesized that surfactant function was dependent on epithelial glycocalyx integrity. Indeed, Hep-I/III–treated mice demonstrated impaired surfactant function, and abnormalities in surfactant processing were noted on ultrastructural analysis with transmission electron microscopy. These included the formation of discrete intra-alveolar edema beyond the resolution limit of light microscopy (visible only after vascular perfusion fixation) and accumulation of lamellar lipid material in the protein-rich liquid film as well as in alveolar macrophages and in capillaries, which may reflect morphologic correlates of increased degradation and removal of secreted surfactant material.

Other cell-surface glycocalyces, including the pulmonary endothelial glycocalyx, derive their biologic functions from their biophysical properties, including sulfation, which provides a negative charge that allows for electrostatic interactions with positively charged proteins (12, 38). We accordingly speculate that epithelial glycocalyx GAGs exert their effects via direct binding to surfactant proteins. We confirmed these findings using heparin affinity chromatography and unbiased mass spectrometry analysis, identifying that HS directly bound SP-A, -B, and -D. Given that SP-B is highly hydrophobic and in vivo is surrounded by phospholipids, it is more likely that SP-A and SP-D, both of which are collectins with known carbohydrate binding domains, are responsible for the binding to epithelial glycocalyx components (39). As GAG shedding correlated with SP-D release into airspace fluid, we speculate that epithelial glycocalyx integrity is necessary for the stabilization of surfactant at the epithelial surface. Although SP-D does not directly bind to surfactant lipids, we speculate that the interaction between SP-D and the glycocalyx may aid in the localization of SP-B within the surfactant hypophase. Lung compliance remained impaired in the absence of cell-surface GAGs even after clearance of airspace GAG fragments, supporting that loss of epithelial glycocalyx integrity (and not the release of soluble factors) induces surfactant dysfunction.

Our data, in which epithelial glycocalyx degradation occurred in only a subset of patients with direct lung injury, underscore that the clinical categorization of “direct” and “indirect” is likely an oversimplification of the multiple and complex mechanisms that contribute to ARDS pathophysiology (40, 41). Additionally, given that critically ill patients are frequently affected by multiple lung-injurious stimuli simultaneously, biomarker-guided identification of the dominant pathophysiologic mechanisms (epithelial-predominant vs. endothelial-predominant) of injury would provide substantial clinical value at the bedside. Although we have demonstrated the feasibility of rapid colorimetric quantification of GAGs in HMEF, additional investigations of the time course of glycocalyx degradation and regeneration in ICU patients (and the ability of airspace GAGs to predict changes in lung mechanics) are warranted. Additionally, analysis of simultaneously collected plasma and HMEF would elucidate the compartment-specific patterns of glycocalyx degradation and their association with ARDS physiology and outcomes. The present study was performed under an IRB-approved waiver of informed consent (necessary for rapid recruitment of an unbiased population of patients in respiratory failure), precluding the collection of plasma samples.

In conclusion, our bedside-to-bench findings demonstrate that alveolar epithelial glycocalyx degradation is sufficient to induce surfactant dysfunction and impaired lung function and thus likely contributes to lung injury pathogenesis and severity. Furthermore, our findings identify a colorimetric assay suitable for point-of-care assessment of glycocalyx integrity. This assay has benefits over other potential assays to quantify MMP expression, including the relative stability of GAGs over proteins and the relative simplicity and cost of the DMMB colorimetric assay ($2 and <1 minute per sample) (32). Shedding of alveolar GAGs could thus be used for predictive enrichment of future ARDS clinical trials in which agents that prevent glycocalyx degradation could be prioritized in subsets of patients who demonstrate elevated HME GAGs by DMMB assay. These efforts promise better personalization of ARDS care, overcoming historic limitations in the management of this highly heterogeneous syndrome.

## Methods

### Reagents.

The HME filters used in this study were the Type I Adult Hygroscopic Condenser Humidifier from AirLife Trach. Heparinase I and III derived from *Flavobacterium heparinum* and chondroitinase ABC were purchased from MilliporeSigma. HS clone F58-10E4 antibody was obtained from AmsBio. Exogenous heparan sulfate (HS) and chondroitin sulfate (CS) were purchased from Galen Laboratories. PR8 influenza was obtained from ATCC.

### Clinical participant phenotyping.

Patients with bilateral infiltrates on post-intubation chest x-ray were designated as having ARDS (Berlin criteria; ref. 16), hydrostatic pulmonary edema, or mixed pulmonary edema by a blinded 2-physician review of clinical data. Participants without bilateral infiltrates were designated as “neither.” Subsequently, patients in the neither category were classified as “airway protection” if they were intubated in the absence of pulmonary disease (e.g., alcohol withdrawal, stroke, seizure, arrhythmia). All others were designated as “other or unclassifiable,” which included a wide range of causes of respiratory failure. Clinical data including demographics, ARDS risk factors, medical comorbidities, ventilator settings, laboratory data, and outcomes were prospectively recorded using the EMR. Participants classified as having ARDS were designated as having “direct lung injury” if their ARDS risk factors included either aspiration or pneumonia.

### RALE scoring.

RALE scoring was performed independently by 2 physicians who were blinded to clinical and biomarker data for all patients with ARDS in our cohort using a previously published protocol (42). Briefly, RALE scores are calculated by dividing the chest radiograph into 4 quadrants (defined vertically by the vertebral column and horizontally by the first branch of the left main bronchus). Each quadrant is then assigned a consolidation score (0 to 4) to quantify the extent of alveolar opacities and a density score (1 to 3) to quantify the density of the opacities (1 = hazy, 2 = moderate, 3 = dense). A final score for each radiograph is calculated by multiplying the consolidation and density scores for each quadrant and then adding the scores of the 4 quadrants to get a final score that ranges from 0 (no infiltrates) to 48 (dense consolidation in >75% of each quadrant).

### Isolation and derivatization of GAGs.

GAGs were isolated and derivatized as previously described with modifications (17, 43). Briefly, HME samples were thawed and mixed well using a vortex mixer. A 200 μL aliquot of each sample was desalted by passing through a 3-kDa-MW-cutoff column and washed twice with liquid chromatography–mass spectrometry–grade (LC-MS–grade) water. In the column, the samples were digested with 1 U each of recombinant heparin lyase I, II, III (pH optima 7.0−7.5) and recombinant chondroitin lyase ABC (pH optimum 7.4) in 350 μL of digestion buffer (50 mM ammonium acetate containing 2 mM calcium chloride, adjusted to pH 7.0), at 37°C for 4 hours. Enzymatic digestion was terminated by removal of the enzymes by centrifugation. Under these reaction conditions, these lyases could completely depolymerize their GAG substrates (in amounts of over 100 μg) into products containing each class of GAG disaccharides. The 3-kDa-filter unit was washed twice with 250 μL LC-MS–grade water, and the filtrates, containing the GAG disaccharide products, were lyophilized overnight. The dried samples were derivatized with 2-aminoacridone (AMAC) by addition of 20 μL of 0.05 M AMAC in DMSO/acetic acid (17:3 vol/vol) and incubation at room temperature for 5 minutes, followed by addition of 20 μL of 0.5 M aqueous NaBH_3_CN and incubation at 45°C for 1 hour. A mixture containing all 17 disaccharide standards prepared at 80–160 nM was similarly AMAC-labeled and used for each analysis as an external standard.

After the AMAC derivatization, the samples and standards underwent a C18 solid-phase extraction (SPE) cleanup to remove the DMSO. The samples and standards were diluted with LC-MS–grade water to reach less than 3% DMSO. The SPE filters were washed twice with 80% acetonitrile (ACN)/0.1% formic acid, followed by 2 washes with 0.1% formic acid. The entirety of the sample was then loaded onto the SPE cartridge. The cartridge was then washed 3 times with 0.1% formic acid. After the final wash, the GAG was eluted with 80% ACN/0.1% formic acid into a clean collection tube. The eluent was transferred into an autosampler vial and evaporated to dryness by speed vacuum concentrator. The samples were reconstituted in mobile phase A (see below), and half of the volume of the sample was used for LC-MS/MS analysis. The rest of the samples were stored in a light-resistant container at 4°C.

### LC-MS/MS analysis.

The samples were analyzed by liquid chromatography–tandem mass spectrometry (LC-MS/MS) as previously described with modification (17). The analytical system was a Dionex LC system composed of pump, sampler, cooling in tandem with an AB Sciex QTRAP 5500. Chromatography was reverse-phase and was performed on a Waters Aquity UPLC BEH-C18 (150 × 1.0, 1.7 μm) with an Aquity UPLC BEH-C18 (5 × 2.1, 1.7 μm) guard column using a gradient elution of mobile phase A (95:5 water/methanol, 1 mM ammonium acetate, pH 9) and mobile phase B (95:5 methanol/water, 1 mM ammonium acetate). The gradient was as follows: 0–15 minutes, 0%–15% B; 15–17 minutes, 15%–35% B; 17.01 minutes, 100% B; 20 minutes, 100% B; 20.01 minutes, 0% B; 23 minutes, 0% B at a flow rate of 0.1 mL/min. The column temperature was held at 45°C, and the injection volume was 18 μL. After every injection, we ran a 20-minute positive run to maintain signal intensity, as the constant addition of sulfate ions will occasionally suppress the signal as the run goes on. The mass spectrometer was operated in electrospray negative ionization mode with multiple reaction monitoring mode. Gas parameters were optimized based on the performance of the standards and were as follows: curtain gas was set to 30 psi, ion spray voltage was set to –4500 V, temperature was set to 400°C, and ion source gas 1 and ion source gas 2 were 30 and 15 psi, respectively. For the compound-dependent parameters, entrance potential and collision cell exit potential were –10 V and –23 V, respectively.

### Quantification of protein biomarkers in HMEF.

Multiplex ELISAs were performed for the quantification of MMP-7, MMP-9, RAGE, Ang2, and SP-D according to the manufacturer’s instructions (Mesoscale Discovery). IgM was quantified in HMEF samples using a commercially available kit (Abcam).

### DMMB assay.

DMMB reagent was prepared using glycine, sodium chloride, and acetic acid as previously described and adjusted to pH 3 (32). DMMB reagent (200 μL) was added to HMEF samples (20 μL) or standards, and the absorbance was measured on a spectrophotometer at 525 nm.

### Murine model of influenza-induced lung injury.

Eight- to 12-week-old male and female C57BL6/J mice (The Jackson Laboratory) were used for these experiments. Influenza (30,000 PFU of PR8 influenza) was delivered via intranasal instillation, and mice were harvested on day 7. At the time of harvest, 3 sequential 1 mL BALs were performed using ice-cold PBS and processed as previously described (8).

### Murine models of alveolar epithelial glycocalyx degradation.

Eight- to 12-week-old male C57BL/6J mice were used in these experiments (The Jackson Laboratory). The alveolar epithelial glycocalyx was selectively degraded in mice by intratracheal injection of Hep-I/III (15 U in 40 μL PBS). An equal concentration and volume of heat-inactivated (20 minutes, 100°C) Hep-I/III were administered to the control group. Before harvests, which occurred at 12, 24, and 72 hours after Hep-I/III treatment, mice were anesthetized with i.p. ketamine (100 mg/kg) and xylazine (15 mg/kg). Once adequate anesthesia was attained, a tracheostomy was performed with an 18 g metal cannula, and mice were connected to a FlexiVent small-animal ventilator (SCIREQ). Respiratory drive was suppressed with succinylcholine (0.24 mg/kg, i.p.) 3 minutes before the assessment of lung mechanics. BAL was performed as described above and was subsequently analyzed with bicinchoninic acid assay (Pierce) and lactate dehydrogenase release assay (CytoTox Non-Radioactive Cytotoxicity Assay, Promega) per manufacturer instructions. GAG levels were determined by LC-MS as described above, and GAG concentrations were corrected for BAL as described above. Immunohistochemistry to visualize the alveolar epithelial glycocalyx was performed on unfixed, inflated, and frozen tissue, in order to preserve epithelial GAGs, as previously described (8). Similar experiments were performed using chondroitinase ABC (2 U in 40 μL PBS), with heat-inactivated enzymes (20 minutes, 100°C) as a negative control. Animals in these experiments were harvested 24 hours after treatment.

### Alveolar to capillary permeability.

Biotinylated BSA (5%) was administered via dose-adjusted intrabronchial administration (20 μL to left lung and 40 μL to right lung) to mice that had been treated with Hep-I/III (15 U) for 24 hours prior. After 1 hour, whole blood was collected by inferior vena cava puncture, and plasma was isolated by centrifugation (1000*g* for 10 minutes). Plasma was transferred to the 10 kDa spin column and centrifuged at 10,000*g* for 10 minutes. The undiluted flow through was analyzed against a standard curve of biotin. Plates were read at 500 nm absorbance.

### RNA sequencing.

RNA was isolated from whole-lung homogenates of mice treated with intratracheal Hep-I/III or HI Hep-I/III (*n* = 3 mice per treatment group) for 24 hours using Qiagen RNeasy Midi kit with an on-column DNase I treatment step per the manufacturer’s instructions. High-quality RNA was submitted to the University of Colorado Cancer Center Genomics and Microarray Core, which prepared indexed single-read mRNA libraries using Nugen Universal mRNA Library Prep Kit. Samples were sequenced with an Illumina HiSEQ 4000. Raw sequencing reads in FASTQ format were adapter-trimmed, aligned, and annotated to the mouse reference genome using Qiagen CLC Genomics Workbench default settings (version 21.0.5). The uniquely mapped transcript matrix was normalized and differential expression was calculated using the DESeq2 R package. *Z* score–transformed heatmaps were generated with the pheatmap R package (Kolde R; Pheatmap: Pretty Heatmaps, R Package Version 1.0.8; 2015; http://CRAN.Rproject.org/package=pheatmap). RNA-sequencing data were deposited to the NCBI’s Gene Expression Omnibus (GEO GSE186705).

### Micro-CT.

Hep-I/III–treated (or HI Hep-I/III–treated) mice were anesthetized as described above. Tracheostomy was performed, and mice were connected to a FlexiVent and placed in the animal bed of a Bruker SkyScan1276 microCT. Extended breath holds were performed at 30, 15, and 0 cmH_2_O. During the breath hold, which was a terminal breath hold, CT scans were taken. The following scanning parameters were used: 70 kV voltage, 200 μA current, 40.5 μm image pixel size, rotation step of 1°, 180° scan, with an aluminum 0.5 mm filter. Scans were reconstructed for analysis following reconstruction with NRecon software from Bruker. Aerated lung volume and Hounsfield units (HU) were calculated using the computer program CTan by Bruker. The CT scan was turned into a binary image, and a threshold was chosen for what represents aerated lung and what represents other tissues. This same threshold was applied to all scans uniformly. Through a series of computer manipulations, the lungs were separated from the rest of the body. The aerated lung volume was then calculated with the software. The HU was also calculated with the software based on a comparison scan of distilled water as a standard value.

### Mouse lung harvest and tissue processing for stereology and electron microscopy.

Mouse lungs were fixed during baseline ventilation (tidal volume = 10 mL/kg, respiratory rate of 150 breaths/min, positive end expiratory pressure = 3 cmH_2_O) through the vasculature to maintain surface tension effects and allow comparison with lung function data as previously described (44). Briefly, bilateral thoracotomy was performed during baseline ventilation, and the pulmonary circulation was then flushed with heparinized saline with 3% 100 kDa dextran. The airway pressure was raised to 30 cmH_2_O and then ramped down to 10 cm H_2_O, the trachea was ligated, and the lungs were then perfused with 1.5% glutaraldehyde, 1.5% paraformaldehyde in 0.15 M HEPES buffer before immersion fixation for at least 2 days. Both the flush and fixative solutions were instilled at a constant pressure of 35 cmH_2_O. Nonpulmonary tissue was dissected away, and the total lung volume was determined using Archimedes’ principle. The lungs were cut to 1.5 mm transverse “slabs,” and randomly selected even- or odd-numbered slabs were then postfixed with 1% OsO_4_ in 0.1 M cacodylate buffer for 2 hours and then overnight in half-saturated aqueous uranyl acetate, dehydrated in a graded acetone series, and embedded in glycol methacrylate (Technovit 7100, Heraeus Kulzer) to avoid tissue shrinkage (44). Slides were stained with toluidine blue for design-based stereology.

Tissue was prepped for electron microscopy by the Electron Microscopy Core Facility at University of Colorado Anschutz Medical Campus. Briefly, 1.5 mm tissue cubes were cut from the slabs that were not used for light microscopy; the sampling location for the cubes was determined using systematic uniform random sampling. The cubes were postfixed with 1% OsO_4_ in 0.1 M cacodylate buffer for 2 hours and then overnight in half-saturated aqueous uranyl acetate, dehydrated in a graded acetone series, and incubated in a 1:1 acetone/araldite solution for 4 hours, then overnight in pure araldite. Samples were embedded in araldite and polymerized at 60°C for 48 hours.

### Design-based stereology.

Stereologic analysis was conducted according to the published standards for lung structure quantification (45). Image acquisition was conducted using an Olympus BX53 with a DP73 camera controlled with NewCast stereology software (Visiopharm). Images were gathered for analysis by the morphometry software via systematic uniform random sampling, an approach that blinds the investigator to image selection, thus removing the potential for bias. A cascade sampling design was used, and all volume fractions were quantified by point counting. The lung parenchymal volume [Vv(par/lung)], defined as areas contributing to gas exchange and excluding airways, vessels outside the septal walls, and peribronchiolar tissue, was analyzed at ×5 magnification with 100% area sampling fraction. The lung parenchymal volume was then subdivided into volume fractions of airspace [Vv(air/par)] and non-air material [Vv(non-air/par)], which was performed at ×20 magnification with 30% area sampling fraction. The non-air material within the parenchyma [Vv(non-air/par)] was then subdivided into volume fractions of septal tissue of open alveoli [Vv(sep,air/par], collapsed septal tissue [Vv(sep,total/par)], and airspace edema [Vv(edema/par)], which was performed at ×40 magnification with 10% area sampling fraction. Collapsed septa were defined as areas in which 2 or more alveolar septal capillaries were directly abutted against each other without intervening airspaces. The volume of each compartment was determined by multiplication of the volume fractions by the volume of the reference space. The surface area per volume available for gas exchange [Sv(alvair/par)] was estimated at ×40 magnification with 10% sampling fraction by counting of line intersections with aerated septal tissue. The gas exchange surface area [S(alvair)] was determined by multiplication of Sv(alvair/par) by the parenchymal volume [V(par)] and the mean septal thickness defined as τ(sep) = 2 V(sep,air)/S(alvair).

### Transmission electron microscopy.

Ultrastructural analysis of Hep-I/III–treated mouse lungs was conducted on a Leo 906 transmission electron microscope (Carl Zeiss) and a Tecnai G2 transmission electron microscope (Thermo Fisher/FEI) and included a total of 11 samples from 3 lungs treated with HI Hep-I/III and 11 samples from 5 lungs treated with Hep-I/III.

### Quantification of surfactant content and biophysical properties.

An aliquot of cell-free BAL from Hep-I/III–treated (and HI Hep-I/III–treated) mice was used to measure the total amount of surfactant, with the remainder centrifuged (40,000*g*, 15 minutes) to separate surfactant subtypes with the supernatant containing small aggregates and the pellet, which was resuspended in 300 μL of 150 mM NaCl, containing the large aggregates as previously described (46). Quantification of the phospholipids in BAL was performed by measurement of the phosphate content of the fluid by the method of Bligh and Dyer (47). For the analysis of the phospholipid composition, the extracted lipids were separated by thin-layer chromatography (TLC) on Silica Gel 60 plates (Merck), using a solvent system consisting of chloroform/methanol/2-propanol/0.25% KCl/triethylamine) (30:9:25:6:18, vol/vol). The TLC plate was sprayed with 0.1% 8-anilino-1-naphthalene sulfonic acid, and the lipid classes were visualized by exposure to ultraviolet light. Each phospholipid class was scraped from the TLC plate and quantified by measurement of phosphate (48).

The biophysical properties of surfactant were assessed by constrained sessile drop (CSD) surfactometry as previously described (49). Briefly, the large-aggregate fractions were resuspended at a phospholipid concentration of 2 mg/mL in buffer (140 mM NaCl, 2.5 mM HEPES, and 1.5 mM CaCl_2_, pH 7.4). Glass beads were added to samples to promote mixing, and samples were heated at 37°C for at least 1 hour before use on CSD. A 9 μL drop of each sample was placed on the CSD pedestal within an environmentally controlled chamber at 37°C and atmospheric humidity. After 2 minutes of adsorption, samples were exposed to 20 repeated compression/expansion cycles on the CSD at a rate of 30 cycles/min. Drop images were recorded during compression/expansion cycles and were analyzed by Axisymmetric Drop Shape Analysis software to obtain an accurate measurement of surface tension. Samples from each mouse had 5 or 6 technical replicates exposed to dynamic compression/expansion cycles.

### Heparin affinity chromatography.

Mouse BAL fluid from male and female mice from the murine influenza experiments described above was pooled (2–4 mice per group) for heparin affinity chromatography. Separation of proteins was achieved using a Hi-Trap Heparin HP column (Cytiva) on an ÄKTA start (Cytiva) liquid chromatography (LC) system attached to a Frac30 (Cytiva) fraction collector. The LC method used a flow rate of 5 mL/min and was performed at 4°C. The following complex gradient was applied after loading of the sample to achieve separation: a 2.5 column volume (CV) wash at the beginning, an increase to 30% mobile phase B (2.0 M Na^+^) over the course of 6 CVs, an increase to 75% mobile phase B over the course of 3 CVs, and lastly a 4 CV long flush at 100% mobile phase B; 1 CV = 5 mL = 1 minute of flow. Analysis was done on UNICORN start 1.0 software (Cytiva). The heparin binding fraction was isolated, and proteomic mass spectrometry was performed on a Q Exactive HF LC-MS (Thermo Fisher Scientific) by the University of Colorado Anschutz Medical Campus Mass Spectrometry Core Facility.

### Statistics.

Statistical analyses of the human participant data were performed in R version 4.0.2 by independent biostatisticians of the University of Colorado School of Public Health Center for Innovative Design and Analysis. Partitioning around medoids (PAM; or k-medoid) clustering was performed to identify patients with high, medium, and low GAG shedding. The 3 individual GAGs (CS, HS, and HA) were used as the input variables after square root transformation to minimize the impact of right skew in a fashion that can adequately handle zero values. Unadjusted relationships between shedding cluster and other covariates (age, sex, race, ethnicity, BMI, and smoking status) were tested using Fisher’s exact tests for categorical variables, and Kruskal-Wallis tests for continuous variables. To assess how shedding relates to age and sex, we modeled (square root–transformed) total shedding as the outcome in a linear regression model using the confounders mentioned above as well as an age-by-sex interaction.

The bivariate associations between GAG shedding and clinical outcomes in patients with ARDS were analyzed using Spearman’s correlation. Additionally, multivariable analyses were performed to control for potentially relevant confounders (age, sex, race, and BMI). A proportional odds ordinal regression model was used for continuous outcomes (P_a_O_2_:F_i_O_2_, duration of mechanical ventilation, ICU length of stay, and hospital length of stay), treating outcomes as ordinal variables (50). Additionally, multiple logistic regression was performed to determine whether GAG shedding was associated with mortality after controlling for the same potentially relevant confounders. The relationship between GAG shedding and DMMB, lung injury biomarkers (RAGE, Ang2, and SP-D), and MMPs was assessed by Spearman’s correlation. Differences were considered statistically significant if *P* was less than 0.05.

Statistical analyses of the murine model data were performed in Prism (GraphPad Software). Data are represented as mean ± SEM. Two-tailed unpaired Student’s *t* test was used to compare 2 groups, and ANOVA with Dunnett’s post hoc testing was used for comparisons involving more than 2 groups. Differences were considered statistically significant if *P* was less than 0.05.

### Study approval.

The Vanderbilt University Medical Center Institutional Review Board approved this minimal-risk study (IRB 121042) under a waiver of informed consent given the minimal risk involved with HME filter exchange. Participants included in this study were enrolled between November 2017 and July 2020. Participants were eligible if they were 18 years of age or older and intubated for less than 24 hours prior to study enrollment. The only exclusion criterion for this study was clinical instability precluding safe filter collection (F_i_O_2_ ≥ 80%, positive end expiratory pressure ≥ 15 cm H_2_O, and/or per staff discretion). Because of biosafety concerns and shortages of personal protective equipment during the SARS-CoV-2 pandemic, no COVID-19 patients were included in this study. Upon enrollment, a clinical research coordinator placed a fresh HME filter in the participant’s ventilator circuit, and this filter was collected after a 4-hour dwell time per published protocols (14). HME filters were transported to the laboratory and centrifuged (2100*g*, 10 minutes, 4°C) to collect condensed fluid. HMEF was then aliquoted and stored at –80°C for further analysis. In a second cohort of patients the HME filters were collected at the first routine filter change rather than at a strict 4-hour dwell time. The Institutional Animal Care and Use Committee of the University of Colorado approved all mouse protocols. We purchased 8- to 12-week-old C57BL/6 wild-type mice from The Jackson Laboratory.

## Author contributions

ANR designed studies, conducted experiments, analyzed data, and wrote the manuscript. SMH, KO, YY, and AMW performed experiments and analyzed data. YJ analyzed data. ML, LAM, NEW, JBM, IZ, SAM, CJLA, KWK, and DRV performed experiments and analyzed data. KCH, CMS, VEK, RAP, and WMK analyzed data. MO, RAWV, and BJS performed experiments and analyzed data. LBW, JAB, and EPS designed studies, analyzed data, and revised the manuscript.

## Supplementary Material

Supplemental data

## Figures and Tables

**Table 1:**
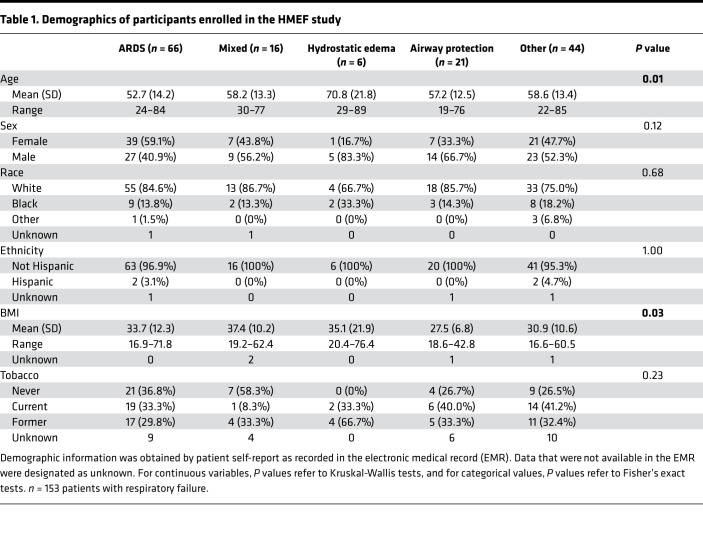
Demographics of participants enrolled in the HMEF study

**Table 2:**
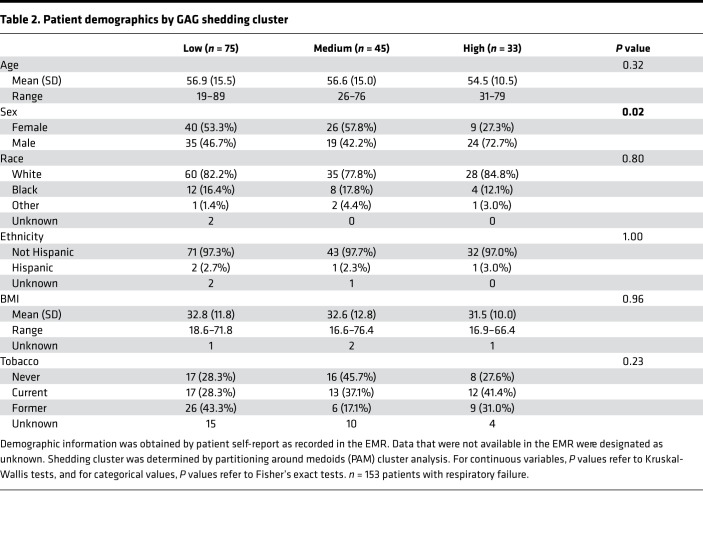
Patient demographics by GAG shedding cluster

**Table 3:**
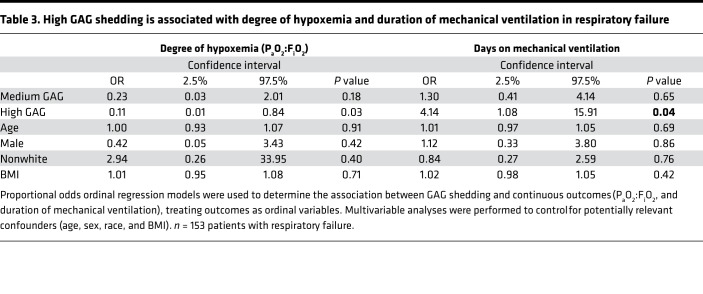
High GAG shedding is associated with degree of hypoxemia and duration of mechanical ventilation in respiratory failure

## Version 1 12/07/2021

In-Press Preview

## Version 2 01/25/2022

Electronic publication
