# Supplementary material for: Alveolar epithelial glycocalyx degradation mediates surfactant dysfunction and contributes to acute respiratory distress syndrome
Source: JCI Insight. 2022 Jan 25;7(2):e154573. doi: 10.1172/jci.insight.154573 (PMC8855818; doi:10.1172/jci.insight.154573)
Supplement: Supplemental data [file jciinsight-7-154573-s100.pdf]

Supplementary Material

Alveolar epithelial glycocalyx degradation mediates surfactant dysfunction and contributes to acute respiratory distress syndrome

Alicia N. Rizzo[1], Sarah M. Haeger[1], Kaori Oshima[1], Yimu Yang[1], Alison M. Wallbank[2], Ying Jin[1,3], Marie Lettau[4], Lynda A. McCaig[5], Nancy E. Wickersham[6], J. Brennan McNeil[6], Igor Zakharevich[7], Sarah A. McMurtry[1], Christophe J. Langouët-Astrié[1], Katrina W. Kopf[8], Dennis R. Voelker[8], Kirk C. Hansen[7], Ciara M. Shaver[6], V. Eric Kerchberger[6], Ryan A. Peterson[1,3], Wolfgang M. Kuebler[9], Matthias Ochs[4], Ruud A.W. Veldhuizen[5], Bradford J. Smith[2,10], Lorraine B. Ware[6], Julie A. Bastarache[6], & *Eric P. Schmidt[1,11].

[1]Division of Pulmonary Sciences and Critical Care Medicine, Department of Medicine, University of Colorado, Aurora, CO, USA. [2]Department of Bioengineering, University of Colorado, Aurora, CO, USA. [3]Department of Biostatistics and Informatics, School of Public Health, University of Colorado, Aurora, CO, USA. [4]Institute of Functional Anatomy, Charité-Universitätsmedizin, Berlin, Germany. [5]Department of Physiology and Pharmacology, Western University, London, Ontario, Canada. [6]Departments of Medicine and Pathology, Microbiology and Immunology, Vanderbilt University, Nashville, TN, USA. [7]Department of Biochemistry and Molecular Genetics, University of Colorado, Aurora, CO, USA. [8]Department of Medicine, National Jewish Health, Denver, CO, USA. [9]Institute of Physiology, Charité-Universitätsmedizin, Berlin, Germany, [10]Division of Pulmonary and Sleep Medicine, Department of Pediatrics, University of Colorado, Aurora, CO, USA. [11]Department of Medicine, Denver Health Medical Center, Denver CO, USA.

Correspondence should be addressed to E.P.S. (eric.schmidt@cuanschutz.edu)

A

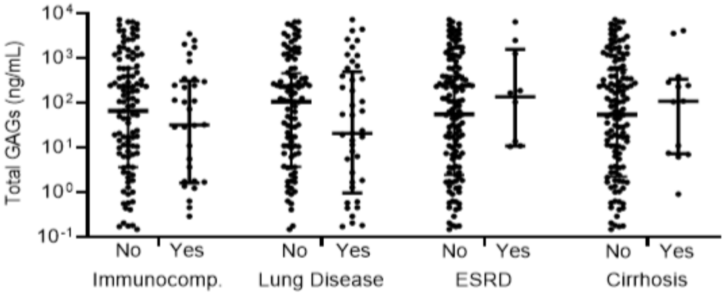

B

Chronic Disease State	n
**Immunocompromised**	**34**
Medication-induced	20
Malignancy	13
Solid Organ Transplant	10
AIDS	2
Other	2
**Lung Disease**	**45**
COPD	32
Asthma	16
Restrictive Lung Disease	2
Other	3
**ESRD**	**10**
**Cirrhosis**	**13**

Supplementary Figure 1: Medical co-morbidities do not impact glycosaminoglycan shedding. **(A)** The amount of glycosaminoglycan (GAG) shedding in heat moisture exchange fluid (HMEF) study patients by chronic disease states including immunosuppression (n = 34), chronic lung disease (n = 45), end stage renal disease (n = 10), and decompensated cirrhosis (n = 13). **(B)** Table identifying the specific medical co-morbidities present within the HMEF study population. Patients being treated with chronic steroids were included as medication-induced immunosuppression. The “other immunocompromised” subjects included one pregnant patient and one patient with pancytopenia. The “other lung disease” subjects included one patient with history of lung transplant, one patient with a lung mass, and one patient with cystic fibrosis. n = 153 subjects with respiratory failure. Comparisons were made by Wilcoxon rank sum tests. There were no statistically significant comparisons to report.

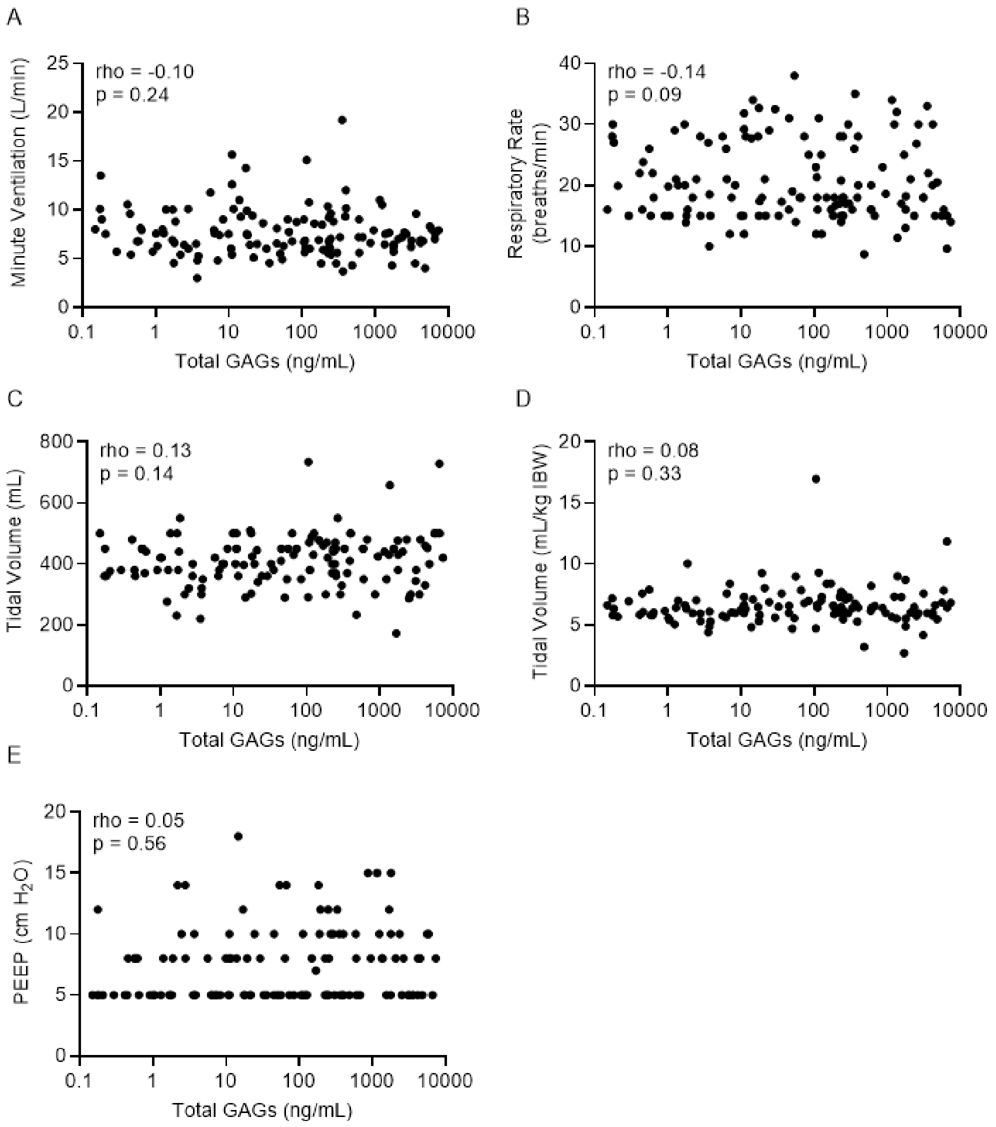

Supplementary Figure 2: Glycosaminoglycan shedding is independent of minute ventilation and ventilator settings. Analysis of total GAG levels compared to **(A)** minute ventilation **(B)** respiratory rate **(C)** tidal volume **(D)** tidal volume per kg of ideal body weight (IBW), and **(E)** positive end expiratory pressure (PEEP). Respiratory rate, tidal volume, PEEP, and minute ventilation was recorded from the subject's ventilator. IBW was calculated by published equations based on height. For males IBW = 0.91 x (Height (cm) – 152.4) + 50 kg and for females IBW = 0.91 x (Height (cm) – 152.4) + 45.5 kg. *n* = 140 subjects with respiratory failure in A-B and 139 subjects in C-E due to missing ventilator data for some subjects (not recorded in electronic medical record).

Spearman correlation and p values are indicated on each graph. There were no statistically significant comparisons to report.

A

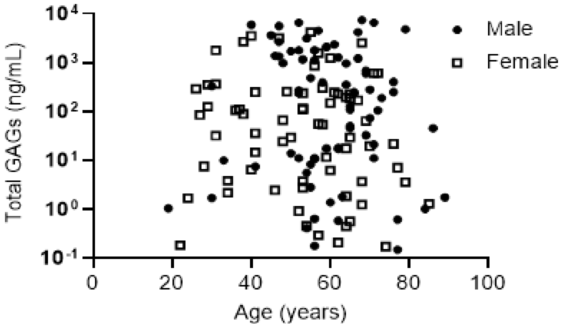

B

	Adjusted interaction effect of age and sex on total GAG shedding			
		Confidence Interval		
	Estimate	2.5%	97.5%	p value
**Intercept**	12.487	2.366	17.607	<0.001
**Male**	9.570	2.250	16.620	0.008
**Age**	-0.097	-0.422	0.227	0.55
**Race: Black**	-4.939	-14.000	4.121	0.28
**Race: Other**	9.098	-11.603	29.799	0.39
**BMI**	-0.121	-0.422	0.180	0.43
**Age:Male**	-0.077	-0.567	0.412	0.75

Supplementary Figure 3: Sex-based differences in GAG shedding do not depend on age. **(A)** Assessment of total GAGs vs. age and stratified by sex. **(B)** Statistical models of total shedding including an interaction between sex and age (centered) were employed to determine if sex-associated differential GAG shedding was dependent on patient's hormonal status. n = 153 subjects with respiratory failure. p values as noted in the table.

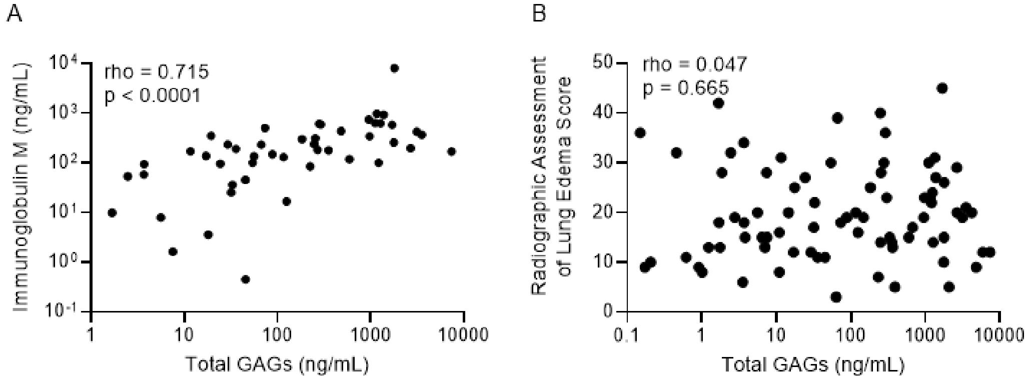

Supplementary Figure 4: Correlation between indices of vascular leak and glycosaminoglycan shedding in respiratory failure. (**A)** HME fluid Immunoglobulin M (IgM) levels are directly related to total GAGs in all-cause respiratory failure. (**B)** Radiographic assessment of lung edema (RALE) scoring is not associated with total glycosaminoglycan shedding. n = 51 patients with respiratory failure in whom sufficient HME fluid was remaining to complete the IgM ELISA. n = 87 patients with bilateral pulmonary infiltrates (ARDS, Hydrostatic Pulmonary Edema, and Mixed ARDS/Hydrostatic) in whom chest imaging was available to compute a RALE score. Rho indicates Spearman correlation. p values as indicated on the graphs.

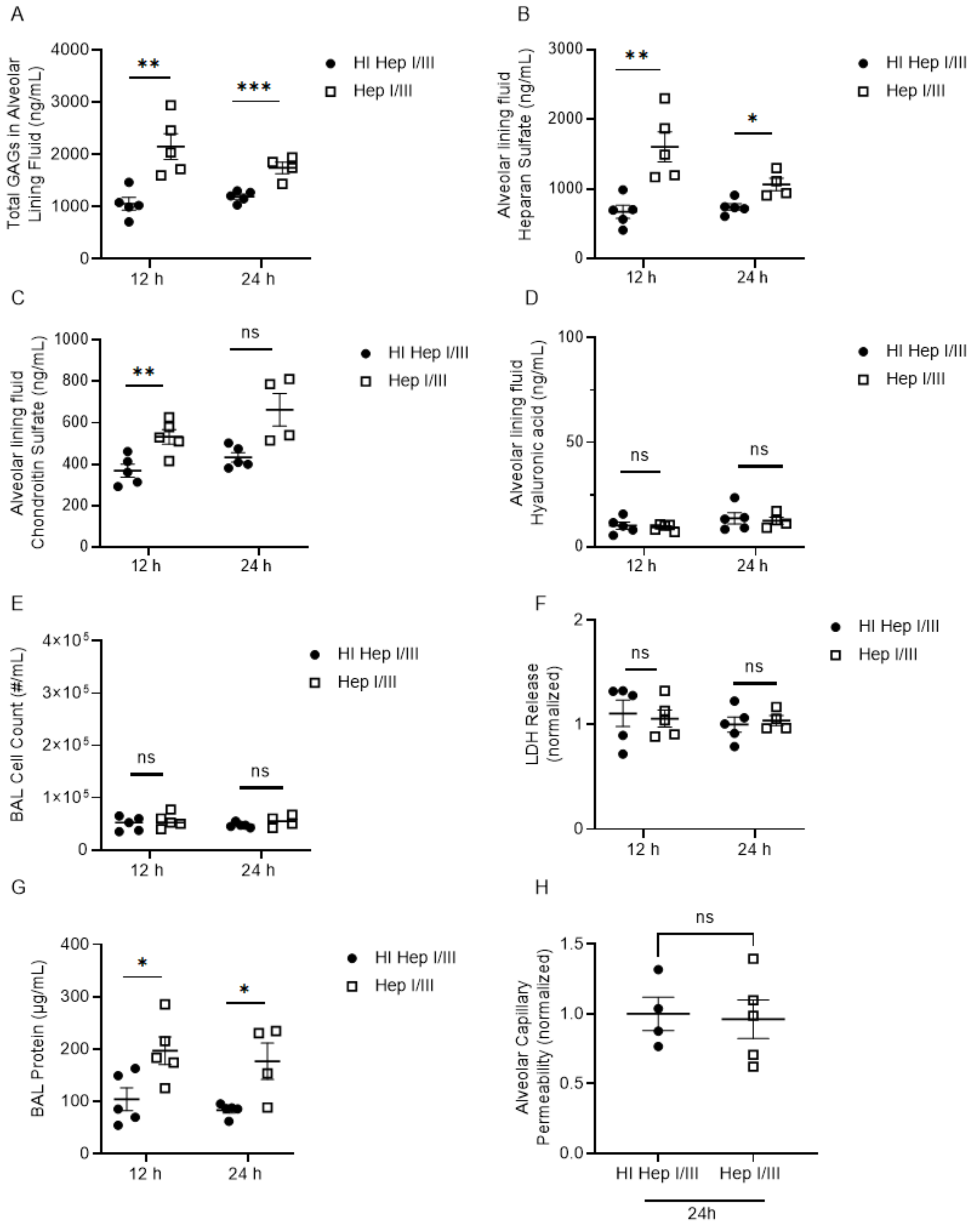

Supplementary Figure 5: Intratracheal heparinase injection degrades the alveolar epithelial glycocalyx without inducing epithelial cellular injury. Male C57BL6J mice were treated with Hep-I/III (15 units, IT) or HI Hep-I/III (15 U, 100 ºC x 25 min) and harvested at 12- and 24-hour time points. **(A)** total GAGs**, (B)** heparan sulfate, **(C)** chondroitin sulfate, and **(D)** hyaluronic acid were measured in the BAL by mass spectrometry. **(E)** BAL cell counts were performed and **(F)** BAL

LDH release (normalized to HI hep I/III 24-hour time point) was quantified. **(G)** BAL protein content was measured by BCA assay. **(H)** Biotinylated albumin was measured in the blood after intratracheal injection to assess the alveolar fluid to blood transport of fluid at 24 hours after Hep-I/III treatment. n = 4-5 mice per group. Comparisons between treatment groups were made by unpaired student's t-test at each timepoint. *$p < 0.05$, **$p < 0.005$, ***$p < 0.0005$. Data are represented as mean +/- s.e.m.

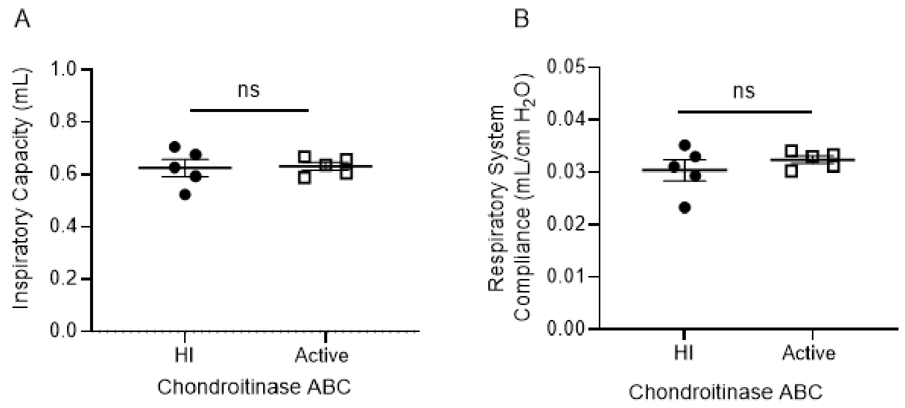

Supplementary Figure 6: Intratracheal chondroitinase injection does not impair lung compliance in mice. Male C57BL6J mice were treated with Chondroitinase ABC (15 units, IT) or HI Chondroitinase ABC (2 U, 100 ºC x 25 min) and harvested at 24-hours. There was no change in the inspiratory capacity **(A)** or respiratory system compliance **(B)** in mice treated with active chondroitinase ABC compared with those treated with HI chondroitinase ABC. n = 4-5 mice per group. Comparisons between treatment groups were made by unpaired student's t test. There are no statistically significant differences to report. Data are represented as mean +/- s.e.m.

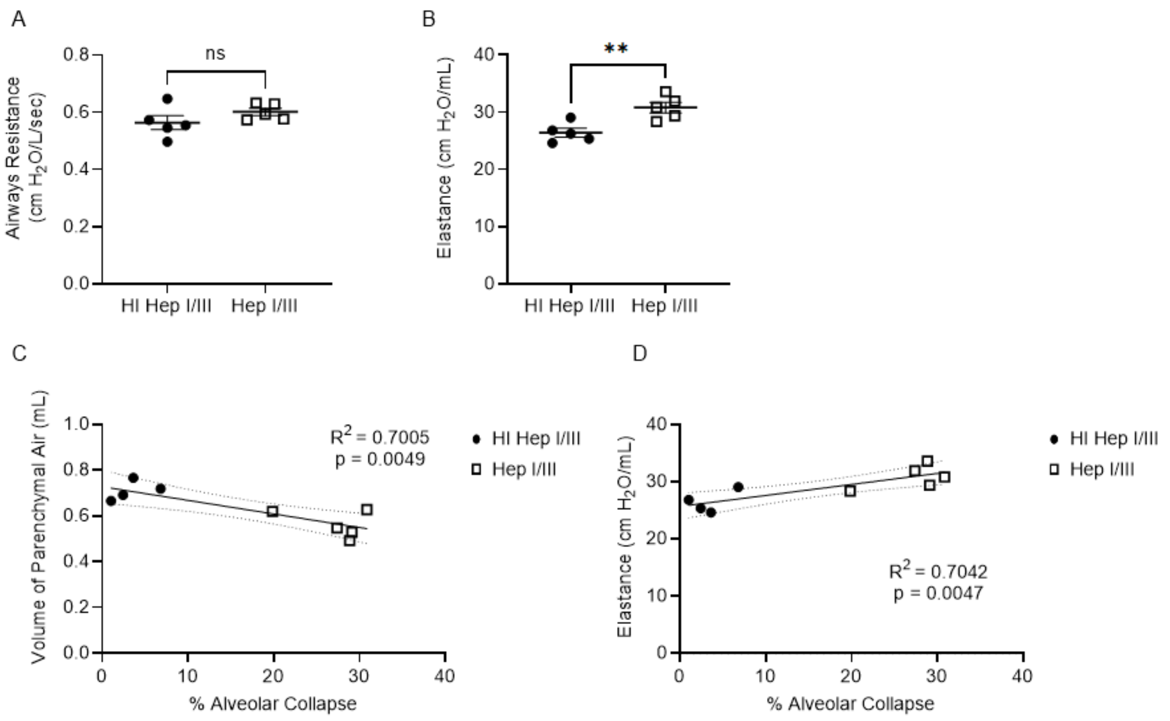

Supplementary Figure 7: Alveolar epithelial glycocalyx degradation mediates impaired lung compliance by inducing microatelectasis. Mice were treated with intratracheal Hep-I/III (15 U in 40 µL, 24 hours) or HI Hep-I/III and **(A)** airways resistance and **(B)** elastance were measured using a FlexiVent. Parenchymal air and percentage of alveolar collapse was assessed by design-based stereology. n = 4-5 mice per group. Comparisons were made by unpaired student's t test. *p < 0.05, **p < 0.005. Data are represented as mean +/- s.e.m. Linear regression was performed to analyze the relationships between **(C)** parenchymal air volume and percentage of alveolar collapse and **(D)** elastance and percentage of alveolar collapse. Correlation data are presented as Pearson r and p values.

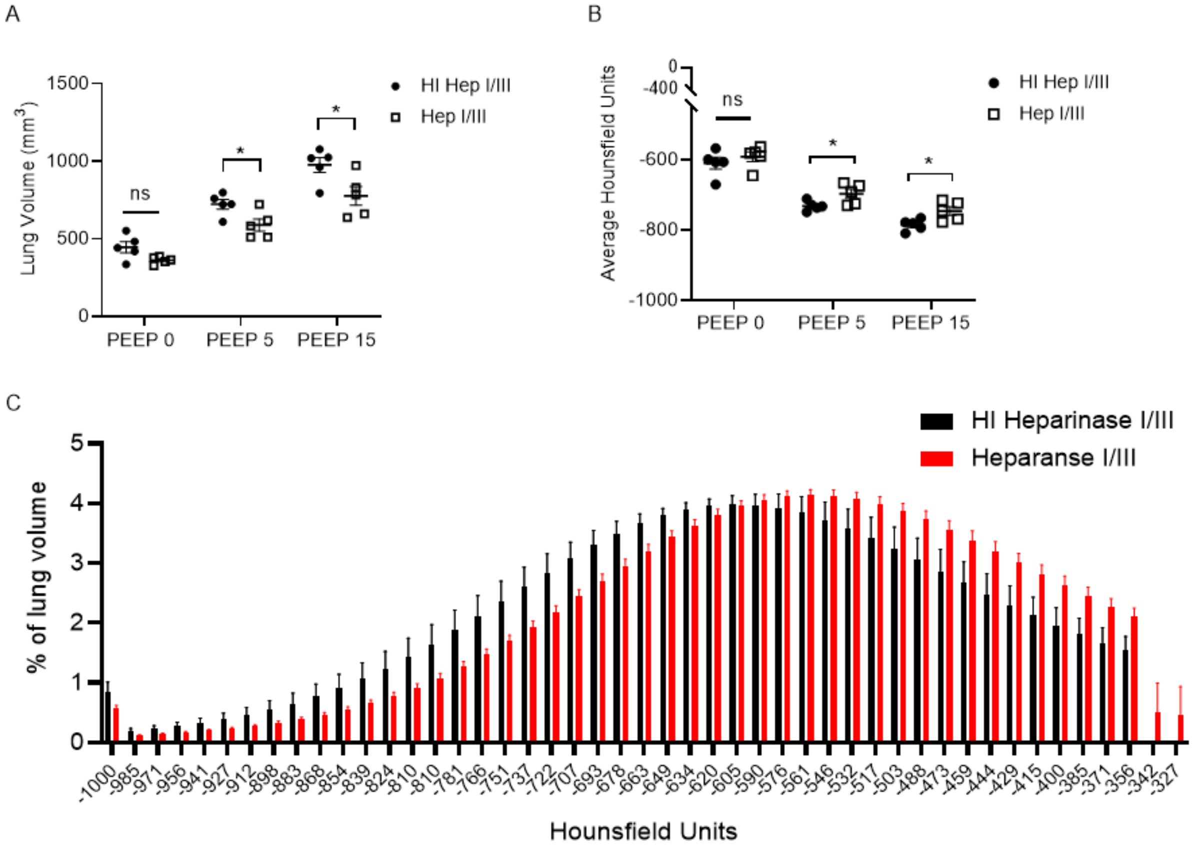

Supplementary Figure 8: Alveolar epithelial glycocalyx degradation causes decreased lung aeration in mice. C57BL6J mice were treated with intratracheal Hep-I/III or HI Hep-I/III (15 U, 100 ºC x 25 min) for 24-hour prior to analysis with microCT. The CT scans were analyzed for **(A)** total lung volume and **(B)** average Hounsfield Units (HU) at 0, 5, and 15 cm H_2O of positive end expiratory pressure. **(C)** Percentage of lung volume at each HU was analyzed with higher HU indicating decreased lung aeration. By convention Air = -1000 HU, Lung = -700 HU, and water = 0 HU. n = 4-5 mice per group. Comparisons between groups were made by unpaired student's t test. **p* <. 0.05. Data are represented as mean +/- s.e.m.

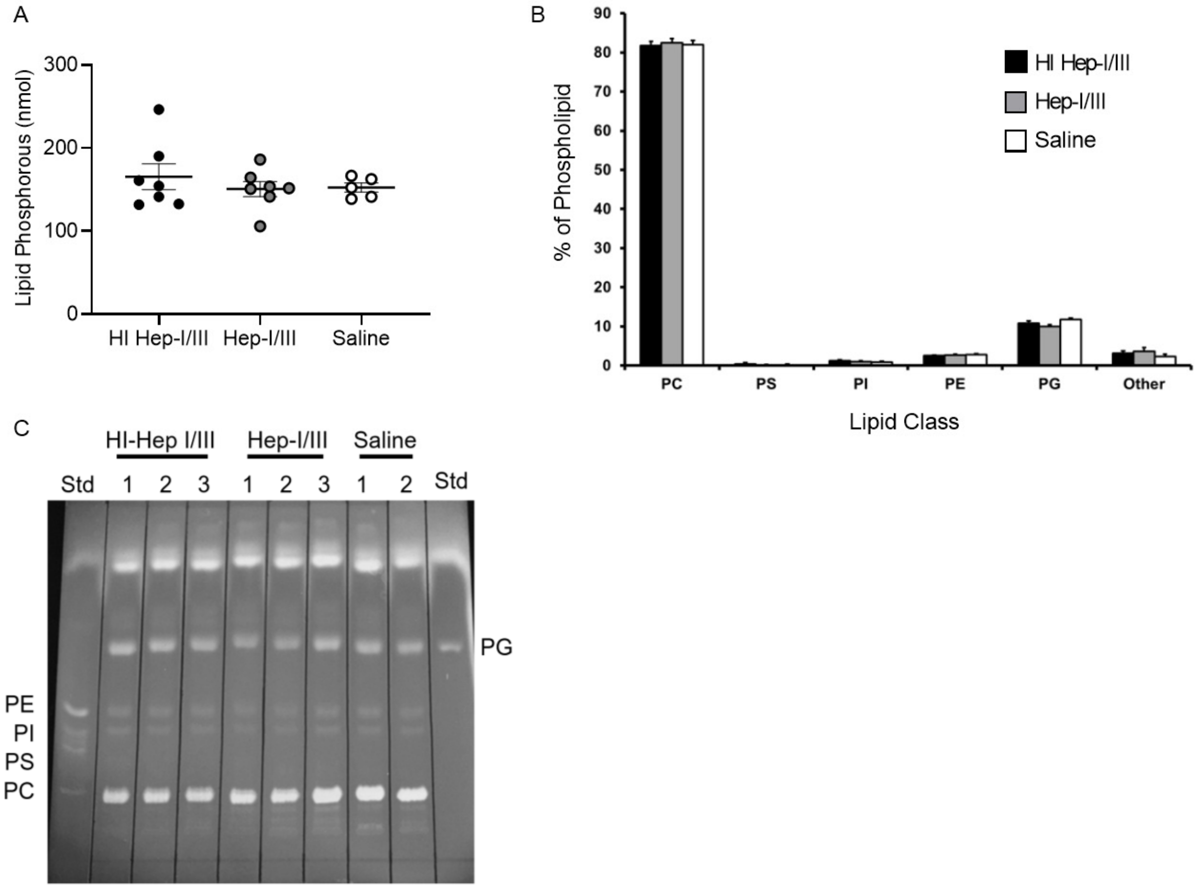

Supplementary Figure 9: Alveolar epithelial glycocalyx degradation does not alter surfactant phospholipid content. The BAL of mice treated with intratracheal Hep-I/III (15 U in 40 μL, 24 hrs), HI Hep-I/III, or saline was analyzed for **(A)** total phospholipid content, using the Bligh and Dryer method, and **(B)** the relative amounts of individual phospholipid species, using thin layer chromatography (TLC). **(C)** Representative image of thin layer chromatography plate. n = 5 mice per group. Comparisons between groups were made using analysis of variance with Dunnett's post-hoc test. There were no significant differences between groups in either analysis. Data presented as mean +/- s.e.m.

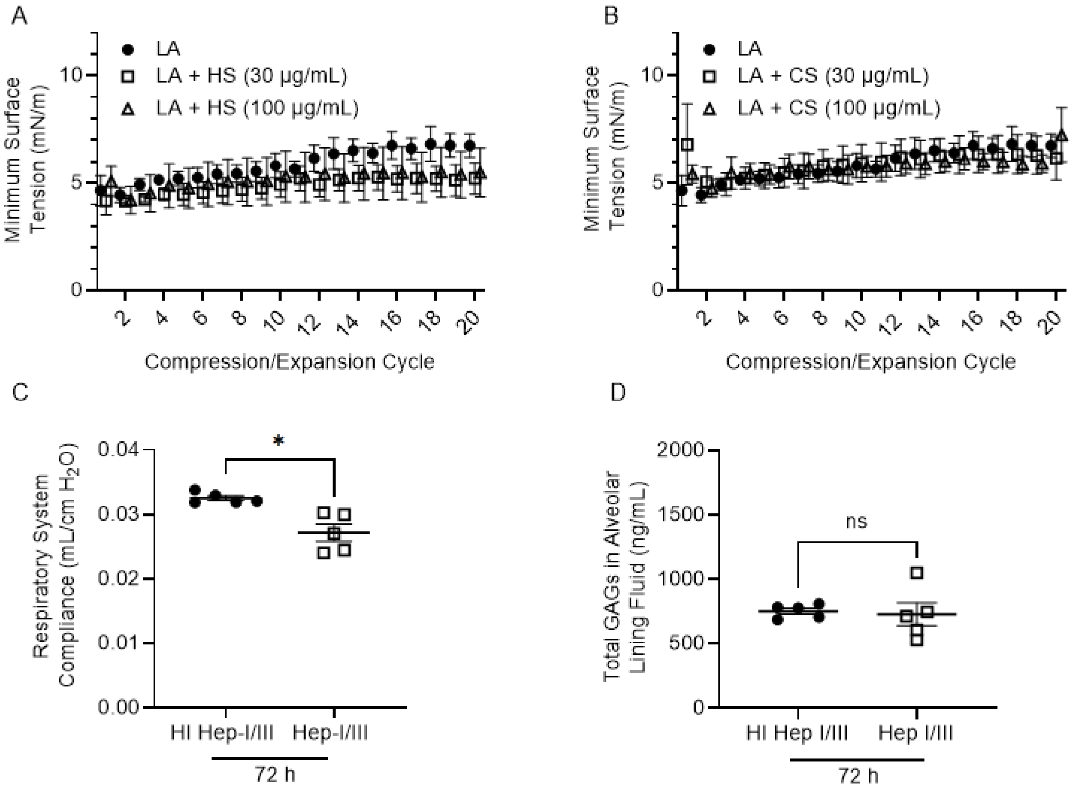

Supplementary Figure 10: Heparinase-I/III induces impaired lung compliance by damaging the epithelial surface glycocalyx rather than the presence of exogenous GAG fragments in the airspaces. **(A)** Heparan sulfate (30 µg/mL, 100 µg/mL) and **(B)** chondroitin sulfate (30 µg/mL, 100 µg/mL) were added to the large aggregate (LA) surfactant subfraction of the BAL of uninjured mice and the minimum surface tension was quantified using constrained sessile drop surfactometry. n = 4-5 mice per group. Comparisons between groups were made using analysis of variance with Dunnett's post-hoc test for multiple comparisons. There were no significant differences between groups to report. In separate experiments, mice were treated with Hep-I/III or HI Hep-I/III (15 U, intratracheal) and were harvested at 72 hours. **(C)** Assessment of the lung compliance at 72 hours post Hep-I/III or HI Hep-I/III treatment **(D),** a timepoint at which the Hep-I/III induced increase in BAL GAGs has resolved. n = 4-5 mice per group. Unpaired student's t test was used for comparisons between groups. *p<0.05, **p<0.005, ***p<0.0005. Data expressed as mean +/- s.e.m.

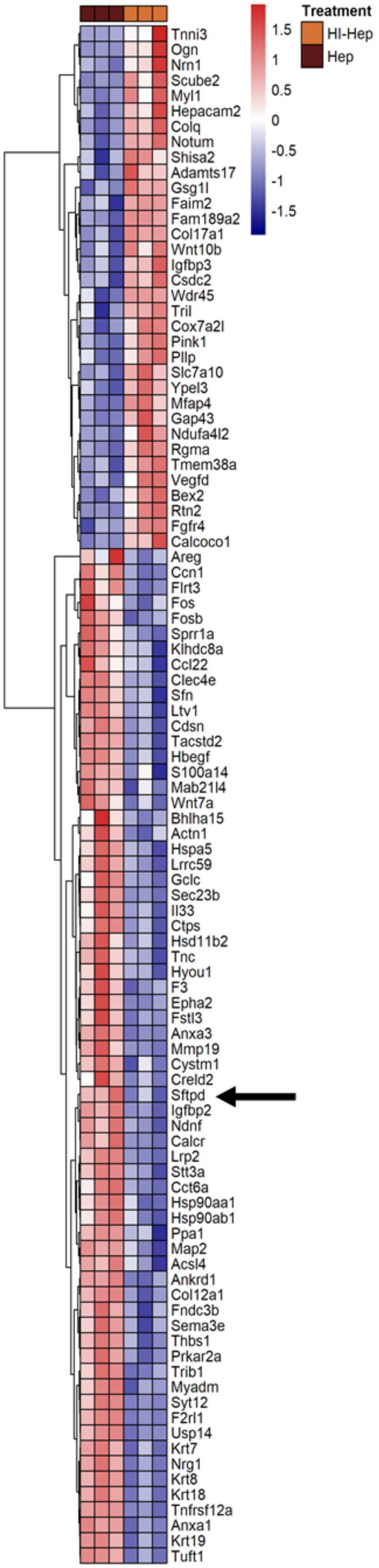
Treatment
HI-Hep
Hep
1.5
1
0.5
0
-0.5
-1
-1.5
Tnni3
Ogn
Nrn1
Scube2
Myl1
Hepacam2
Colq
Notum
Shisa2
Adamts17
Gsg1l
Faim2
Fam189a2
Col17a1
Wnt10b
Igfbp3
Csdc2
Wdr45
Tril
Cox7a2l
Pink1
Pllp
Slc7a10
Ypel3
Mfap4
Gap43
Ndufa4l2
Rgma
Tmem38a
Vegfd
Bex2
Rtn2
Fgfr4
Calcoco1
Areg
Ccn1
Flrt3
Fos
Fosb
Sprr1a
Klhdc8a
Ccl22
Clec4e
Sfn
Ltv1
Cdsn
Tacstd2
Hbegf
S100a14
Mab21l4
Wnt7a
Bhlha15
Actn1
Hspa5
Lrrc59
Gclc
Sec23b
Il33
Ctps
Hsd11b2
Tnc
Hyou1
F3
Epha2
Fstl3
Anxa3
Mmp19
Cystm1
Creld2
Sftpd
Igfbp2
Ndnf
Calcr
Lrp2
Stt3a
Cct6a
Hsp90aa1
Hsp90ab1
Ppa1
Map2
Acsl4
Ankrd1
Col12a1
Fndc3b
Sema3e
Thbs1
Prkar2a
Trib1
Myadm
Syt12
F2rl1
Usp14
Krt7
Nrg1
Krt8
Krt18
Tnfrsf12a
Anxa1
Krt19
Tuft1

Supplementary Figure 11. Heparinase-I/III induces upregulation of surfactant protein D without affecting expression of other surfactant proteins. Whole lung homogenates of mice treated with HI Hep-I/III or Hep-I/III were analyzed by RNA sequencing and a heatmap was constructed to demonstrate the genes that are differentially regulated after Hep-I/III treatment. n = 3 mice per group.

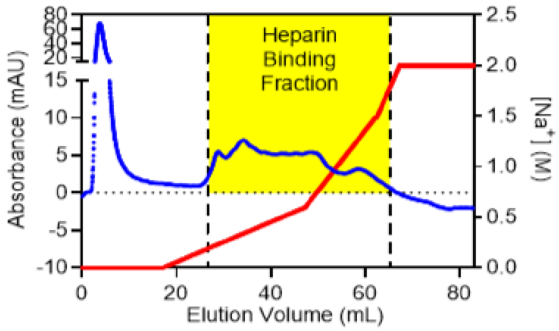

Supplementary Figure 12: Surfactant proteins bind to heparan sulfate in the BAL of mice. Pooled bronchoalveolar lavage fluid from male mice that were harvested at 7 day of influenza infection (30,000 plaque forming units, intranasal) was passed through a heparin affinity column (GE Healthcare), and proteins were eluted from the column with increasing sodium (0–2 M). The heparin binding fractions underwent unbiased proteomic mass spectrometry to identify BAL proteins that bind to heparin. n = 4 mice.
